# Supplementary material for: Differential sensing with arrays of de novo designed peptide assemblies
Source: Nat Commun. 2023 Jan 24;14:383. doi: 10.1038/s41467-023-36024-y (PMC9873944; doi:10.1038/s41467-023-36024-y)
Supplement: Supplementary file 1 — Supplementary Information [file 41467_2023_36024_MOESM1_ESM.pdf]

***Supplementary Information** for*

Differential sensing with arrays of *de novo* designed peptide assemblies

William M. Dawson[1,†,*], Kathryn L. Shelley[1,2,†], Jordan M. Fletcher[1,3], D. Arne Scott[1,2,3], Lucia Lombardi[1,4,5], Guto G. Rhys[1,6,7], Tania J. LaGambina[3], Ulrike Obst[3], Antony J. Burton[1,8], Jessica A. Cross[1,2], George Davies[1], Freddie J.O. Martin[1], Francis J. Wiseman[1], R. Leo Brady[2], David Tew[9], Christopher W. Wood[1,2,10,*] and Derek N. Woolfson[1,2,4,*]

[1]School of Chemistry, University of Bristol, Cantock's Close, Bristol BS8 1TS, U.K.
[2]School of Biochemistry, University of Bristol, Medical Sciences Building, University Walk, Bristol BS8 1TD, U.K.
[3]Rosa Biotech, Science Creates St Philips, Albert Road, Bristol, BS2 0XJ, U.K.
[4]BrisSynBio, University of Bristol, School of Chemistry, Bristol BS8 1TS, U.K.
[5]Department of Chemical Engineering, Imperial College London, London SW7 2AZ, U.K.
[6]Department of Biochemistry, University of Bayreuth, Universitätsstraße 30, 95447 Bayreuth, Germany
[7]School of Chemistry, Cardiff University Main Building, Park Place, Cardiff CF10 3AT, U.K.
[8]AstraZeneca, 35 Gatehouse Drive, MA 02451, United States
[9]GlaxoSmithKline (GSK), Gunnels Wood Rd, Stevenage SG21 2NY, U.K.
[10]School of Biological Sciences, University of Edinburgh, Roger Land Building, Edinburgh EH9 3JQ, U.K.

*Correspondence should be addressed to: w.dawson@bristol.ac.uk, chris.wood@ed.ac.uk and D.N.Woolfson@bristol.ac.uk

† Contributed equally

Supplementary Note

A robust data analysis pipeline to analyze αSA performance

The αSA technology was developed to enable the analysis of multiple sample types, before subsequent optimisation if required. Accordingly, an αSA data analysis pipeline was designed (written in Python) that could apply a range of machine learning (ML) algorithms to a variety of potential datasets using established best practices[1]. A total of 6 ML algorithms were applied to each dataset in an automated fashion with the aim of estimating the performance that could be achieved in the future, and to allow more tailored approaches to be developed for bespoke applications as required. The data analysis pipeline is designed to be as generic as possible rather than pre-selecting a specific ML algorithm to use. This includes checkpoints (detailed below) where the user must analyse the outputs with regards to their dataset before moving to the next step or choosing subsequent variables to use. This removes any "black box" approach as the user has full control of how the dataset is treated in the pre-processing and ML analysis.

Pre-processing was applied to the raw fluorescence αSA array data before ML analysis (Supplementary Figure 10). In stage one, data parsing converted the raw data inputs of the fluorescence readings into dataframe format. Readings were min-max scaled relative to the DPH, "DPH + analyte" and "αHB + DPH" readings on the same plate using Equation 1. Technical repeats of the same analyte – which could be on the same plate or spread across multiple different plates – were then averaged by calculating the median reading for each αHB in the αSA. At that stage, the individual data points and median αSA fingerprints were outputted for visual inspection. Finally, outliers from automated liquid-handling errors were identified using a generalized extreme Studentized deviate (ESD) test[2, 3] and removed before the final datasets were taken into ML analysis.

The αSA ML pipeline (Supplementary Figure 11) trains 6 different classifiers that vary in complexity in addition to two dummy classifiers: K-nearest neighbors[4, 5], Gaussian Naïve Bayes, linear discriminant analysis (LDA), support vector classification (SVC) with either a linear kernel or a radial basis kernel[6], and AdaBoost[7]. The dummy classifiers randomly assign an output class label – and hence mimic random guessing – either by predicting the most frequent class for every sample ("popular") or by scrambling the true labels ("stratified"). By spot checking multiple models, users are able to select the most suitable algorithm for their application. The ML algorithms have been implemented using the open source Python package scikit-learn[8].

To overcome limitations in the amount of data available for training and testing each model, stratified k-folds cross-validation[9] (CV) was employed. Stratified k-folds cross-validation splits a dataset into k subsets, with each subset containing approximately the same relative number of samples of each analyte class as the complete dataset. In each fold, one subset formed the test set while the remaining subsets were merged into the training set, and this was repeated k times. Thus, each subset was used as the test set once, and the overall accuracy was calculated from the mean average ± standard deviation across all k-folds. However, some ML algorithms in the αSA ML pipeline (*e.g.,* SVC) have associated hyperparameters that require tuning, calling for three independent datasets: a training dataset, a validation dataset (hyperparameter tuning) and a test dataset (algorithm selection). In the αSA ML pipeline, two nested CV loops were used (Supplementary Figure 11). The outer loop splits the data into k_1 subsets, with one subset selected to be the test set. The remaining subsets were merged and the second loop divided these data into k_2 subsets, with one subset as the validation set and the remaining subsets merged into the training dataset. This avoided overfitting of the ML algorithms.

For all datasets except the tea, k was set to 5 in both the inner and outer CV loops. For the tea, k was set to 10 in the outer loop and to 9 in the inner loop, in order that each validation/test set comprised all fingerprints measured for one brand of each of the three tea classes. This ensured that, across both the inner and outer CV loops, model performance was always assessed using tea brands that had not previously been seen during model training.

Throughout the αSA ML pipeline, users are required to analyze the outputs and select the subsequent best course of action. Accordingly, the αSA and αSA ML pipeline can be applied to differentiate a wide range of analytes and the resulting data that is generated.

Individual αHB importance can be determined for each application

The αSA used here consisted of 46 αHBs in four different groups: hydrophobic channels, polar mutants, charged mutants and aromatic mutants. However, the majority of these αHBs were similar and differed in a single residue per peptide chain. Therefore, it was possible that different αHBs provided similar information in the αSA outputs, or noise if the analyte did not interact with the reporter dye in the channel. Therefore, feature correlation coefficients (Spearman's rank) were calculated for each classification problem to visualise the classes/subsets of αHBs with high or low correlation coefficients. Where appropriate, the αSA ML pipeline employed methods to determine feature importance of the αHBs in each classification problem (Supplementary Figure 10)[8]. This served the purpose of removing any "redundant" αHBs that provided the same information or added noise to the model. This increased the accuracy of the model and/or reduced compute needed to train the ML algorithms. In addition, removing unnecessary αHBs will allow larger numbers of fingerprints to be collected on each multi-well plate, increasing the robustness of the measurements and reducing overall resources and cost in the future. These are all important considerations for biotechnological applications.

For each dataset, a 5x2 CV F-test (Supplementary Figure 11)[10, 11] was used to test whether the best model (as assessed by accuracy/F1 score) trained using the full αSA (46 αHBs) performed significantly better than the random guessing of the dummy classifiers. A 5x2 CV F-test was also used to compare whether the performance of the best model trained using a reduced number of features differed significantly from that of the best model trained on the full αSA.

Three feature selection methods are implemented in the αSA pipeline: KBest analysis, an ExtraTrees classifier, and permutation analysis. KBest analysis (which in our pipeline calculates the ANOVA F-value between the readings measured for each barrel) is a univariate method *i.e.* it calculates the relationship between each feature (αHB) and the output, and therefore assumes each feature is independent. The sequence and structural similarity of the αHBs in the αSA make this unlikely. Nonetheless, KBest analysis identified the αHBs that provided the most/least signal with regards to the output.

The ExtraTrees classifier trains multiple decision trees on a random subset of data and the results are averaged to make a prediction. Importance scores are calculated as the average increase in purity achieved when using a particular feature to split the data across all trees in which that feature is included (*i.e.* the Gini importance score of the feature). Whilst correlations between the included αHBs are reflected in the scores, as more trees incorporating different subsets of αHBs are included in the average, feature correlations have less of an effect on the importance scores. Thus, correlation between αHBs had little effect on the importance scores in this case as the number of trees was 100 and the number of bootstrap repeats numbered 1000.

In permutation analysis, feature importance scores for each αHB were calculated as the difference between two ML models. The first model was trained with the original dataset, the second trained on a dataset in which the data points of the specific αHB were randomly permuted. As such, this analysis took feature correlations into account by measuring the unique information that a particular αHB provided in the context of all available αHBs.

KBest and permutation analysis were used in the training of the ML algorithms and thus the reduction of features in the αSA for each classification problem. ExtraTrees is an intermediate method compared to the other two feature importance analysis approaches when considering assumptions about feature independence. Additionally, the ExtraTrees classifier selected similar features (αHBs) as the other two methods (Supplementary Fig. 24). Therefore, to optimize the speed and resources required by the αSA ML pipeline, an ExtraTrees classifier was not applied in the feature reduction stage. However, the αSA pipeline has been designed to enables users to choose which feature importance methods to apply for their specific application in the future.

Finally, to limit the likelihood that subsets of αHBs were identified as important by random chance—*i.e.*, for a specific dataset rather than an entire population—the αSA ML pipeline included feature selection on both the whole dataset, and the training set alone within the nested CV. Results of both methods were then compared in the pipeline to confirm that similar αHBs were chosen. This compromise, rather than performing feature selection on a single training dataset for instance, was made due to the relatively small size of datasets that proof-of-concept biosensor studies typically obtain, allowing the number and class of αHBs to be tailored for a specific classification problem at an early stage.

Supplementary Table 1. Sedimentation velocity AUC fitting statistics for the new αHB designs in this study.

Peptide ID	$\bar{v}$[1] (cm^3 g^{-1})	Fitted Mass[2] (95% confidence, 3 SF)	f/f_0[3]	s[4] (S)	$s_{20,w}$[5] (S)
4	0.772	18200	1.238	1.604	1.673
5	0.757	18200	1.251	1.704	1.774
6	0.753	22600	1.300	1.932	2.010
7	0.772	18300	1.200	1.657	1.729
8	0.763	22300	1.317	1.806	1.882
9	0.756	17100	1.224	1.679	1.747
12	0.777	20600	1.302	1.618	1.689
17	0.768	21500	1.295	1.722	1.795
18	0.768	18200	1.233	1.636	1.706
19	0.761	19400	1.176	1.856	1.933
20	0.751	23400	1.300	1.991	2.072
21	0.748	19600	1.208	1.928	2.006
23	0.754	21400	1.215	1.979	2.059
24	0.780	19500	1.124	1.772	1.850
25	0.767	20000	1.213	1.782	1.858
26	0.769	20300	1.248	1.738	1.812
27	0.761	20000	1.205	1.849	1.926
28	0.775	16900	1.228	1.519	1.584
29	0.765	19500	1.299	1.656	1.725
30	0.756	23800	1.500	1.707	1.777
31	0.756	22400	1.423	1.725	1.795
32	0.775	24900	1.362	1.807	1.852
33	0.756	18800	1.293	1.690	1.760
34	0.757	20600	1.182	1.955	2.036
37	0.775	19200	1.257	1.616	1.685
38	0.791	18100	1.264	1.416	1.482
39	0.774	18300	1.317	1.496	1.561
40	0.771	19900	1.171	1.807	1.885
41	0.770	22700	1.212	1.792	1.869
43	0.752	22400	1.287	1.949	2.028
44	0.770	19300	1.193	1.750	1.825
45	0.771	18700	1.126	1.804	1.882
46	0.770	22700	1.300	1.784	1.861

[1] Partial specific volume calculated using Sednterp (http://rasmb.org/sednterp/)
[2] Mass quoted to 3 significant figures
[3] Best-fit frictional ratio
[4] Sedimentation coefficient
[5] Normalized sedimentation coefficient in water at 20 °C

Supplementary Table 2. Crystallisation conditions for the new X-ray crystal structures determined in this study

**Peptide systematic name**[1]	**αHB ID**	**Crystallisation condition**[2,3]
CC-Type2-$[L_aI_d]_4$-L14A	4	50 mM sodium cacodylate, 20% MPD and 2.5% PEG 8000, pH 6.5
CC-Type2-$[L_aI_d]_4$-I24A	7	50 mM TRIS and 10% v/v ethanol, pH 8.5
CC-Type2-$[M_aI_d]_4$	9	250 mM ammonium sulfate and 50 mM MES, pH 6.5
CC-Type2-$[Q_gL_aI_d]_4$	15	50 mM sodium acetate, 1% w/v PEG 4000, pH 5.0
CC-Type2-$[L_aI_d]_4$-I17C	17	400 mM potassium sodium tartrate tetrahydrate and 50 mM sodium HEPES, pH 7.5
CC-Type2-$[L_aI_d]_4$-L21N-I24N	21	100 mM magnesium chloride hexahydrate, 50 mM TRIS and 1.7 M 1,6-hexanediol, pH 8.5
CC-Type2-$[L_aI_d]_4$-I24N	25	50 mM BICINE and 5% v/v MPD, pH 9
CC-Type2-$[L_aI_d]_4$-I24S	26	100 mM sodium citrate tribasic dihydrate, 50 mM sodium cacodylate and 15% v/v 2-propanol, pH 6.5
CC-Type2-$[L_aI_d]_4$-I17K-W19Φ	29	100 mM sodium HEPES, 0.1 M NaCl and 10% v/v 2-propanol, pH 7.5
CC-Type2-$[L_aI_d]_4$-L21K	32	100 mM sodium citrate tribasic dihydrate, 50 mM sodium HEPES and 10% v/v 2-propanol, pH 7.5
CC-Type2-$[L_aI_d]_4$-L7Y	41	100 mM ammonium formate and 10% w/v PEG 3350
CC-Type2-$[L_aI_d]_4$-L28Y	46	50 mM sodium HEPES, 5% w/v PEG 8000 and 4% v/v ethylene glycol, pH 7.5

[1] Φ 4-Bromo-phenylalanine
[2] Dispensed concentrations based on 1:1 dilution with peptide solution
[3] HEPES - 2-[4-(2-hydroxyethyl)piperazin-1-yl]ethanesulfonate; TRIS - 2-amino-2-(hydroxymethyl)propane-1,3-diol; MDP - 2-methyl-2,4-pentanediol; MES - 2-morpholinoethanesulfonic acid; BICINE - 2-(bis(2-hydroxyethyl)amino)acetic acid

Supplementary Table 3. Model parameters and results for the classification of amino acids by the αSA

Algorithm[1]	Features[2]	Accuracy (%)	Recall (%)	Precision (%)	F1 score (%)
Dummy classifier (popular)	10	18 ± 6	18 ± 6	3 ± 2	6 ± 3
Dummy classifier (stratified)		7 ± 10	7 ± 10	11 ± 16	8 ± 12
K-neighbors classifier		58 ± 9	58 ± 9	60 ± 9	56 ± 9
Gaussian Naïve Bayes		69 ± 16	69 ± 16	73 ± 20	69 ± 17
LDA		56 ± 16	56 ± 16	55 ± 20	53 ± 17
SVC (linear)		64 ± 9	64 ± 9	62 ± 12	60 ± 9
SVC (rbf)		51 ± 13	51 ± 13	54 ± 9	50 ± 10
AdaBoost classifier		36 ± 12	36 ± 12	33 ± 19	31 ± 12

[1] Feature selection method: Permutation analysis. LDA – linear discriminant analysis. SVC – support vector classification. Nested stratified k-folds cross validation: k=5 (inner and outer loops)
[2] Two-sided 5x2 CV F-test p-values: full and reduced feature αSA = 0.60 (no significant difference), full αSA and dummy classifier = 0.001 (full αSA significantly better performance)

Supplementary Table 4. Model parameters and results for the classification of fatty acids by the αSA

Algorithm[1]	Features[2]	Accuracy (%)	Recall (%)	Precision (%)	F1 score (%)
Dummy classifier (popular)	2	22 ± 0	22 ± 0	5 ± 0	8 ± 0
Dummy classifier (stratified)		7 ± 6	7 ± 6	7 ± 6	7 ± 6
K-neighbors classifier		100 ± 0	100 ± 0	100 ± 0	100 ± 0
Gaussian Naïve Bayes		100 ± 0	100 ± 0	100 ± 0	100 ± 0
LDA		91 ± 9	91 ± 9	96 ± 4	91 ± 9
SVC (linear)		93 ± 6	93 ± 6	94 ± 8	92 ± 7
SVC (rbf)		100 ± 0	100 ± 0	100 ± 0	100 ± 0
AdaBoost classifier		78 ± 8	78 ± 8	73 ± 11	73 ± 9

[1] Feature selection method: K-Best analysis. LDA – linear discriminant analysis. SVC – support vector classification. Nested stratified k-folds cross validation: k=5 (inner and outer loops)
[2] Two-sided 5x2 CV F-test p-values: full and reduced feature αSA = 0.38 (no significant difference), full αSA and dummy classifier = 0.0003 (full αSA significantly better performance)

Supplementary Table 5. Model parameters and results for the classification of carbohydrates by the αSA

Algorithm[1]	Features[2]	Accuracy (%)	Recall (%)	Precision (%)	F1 score (%)
Dummy classifier (popular)	4	21 ± 1	21 ± 1	4 ± 1	7 ± 1
Dummy classifier (stratified)		19 ± 4	19 ± 4	29 ± 4	22 ± 4
K-neighbors classifier		46 ± 23	46 ± 23	44 ± 23	43 ± 22
Gaussian Naïve Bayes		40 ± 15	40 ± 15	37 ± 14	37 ± 14
LDA		59 ± 20	59 ± 20	57 ± 25	56 ± 22
SVC (linear)		52 ± 25	52 ± 25	50 ± 30	48 ± 26
SVC (rbf)		61 ± 23	61 ± 23	61 ± 29	58 ± 25
AdaBoost classifier		41 ± 13	41 ± 13	26 ± 17	30 ± 17

[1] Feature selection method: KBest analysis. LDA – linear discriminant analysis. SVC – support vector classification. Nested stratified k-folds cross validation: k=5 (inner and outer loops)
[2] Two-sided 5x2 CV F-test p-values: full and reduced feature αSA = 0.029 (reduced feature αSA significantly better performance than the full αSA), reduced αSA and dummy classifier = 0.0001 (reduced αSA significantly better performance)

Supplementary Table 6. Commercial tea brands used in this study

**Tea samples**					
**Number**	**Brand**	**Number**	**Brand**	**Number**	**Brand**
Black 1	Clipper	Green 1	Clipper	Grey 1	Asda
Black 2	Diplomat	Green 2	Diplomat	Grey 2	Clipper
Black 3	Dragonfly Tea	Green 3	Double Dragon	Grey 3	Co-op
Black 4	PG Tips	Green 4	Dragonfly Tea	Grey 4	Devonshire Tea
Black 5	Pukka	Green 5	Holland & Barrett	Grey 5	Diplomat
Black 6	Sainsbury's Gold	Green 6	Joe's Tea Co	Grey 6	Joe's Tea Co
Black 7	Tesco	Green 7	Qi	Grey 7	Marks & Spencer
Black 8	Tetley	Green 8	Sainsbury's	Grey 8	Pukka
Black 9	Twinings	Green 9	Tetley	Grey 9	Tesco
Black 10	Yorkshire Tea	Green 10	Twinings	Grey 10	Twinings

Supplementary Table 7. Model parameters and results for the classification of different teas by the αSA

**Algorithm**[1]	**Features**[2]	**Accuracy (%)**	**Recall (%)**	**Precision (%)**	**F1 score (%)**
Dummy classifier (popular)	4	33 ± 2	33 ± 2	11 ± 1	16 ± 1
Dummy classifier (stratified)		27 ± 3	27 ± 3	23 ± 3	25 ± 3
K-neighbors classifier		82 ± 13	82 ± 13	83 ± 12	82 ± 13
Gaussian Naïve Bayes		79 ± 16	79 ± 16	82 ± 15	78 ± 16
LDA		84 ± 14	84 ± 14	86 ± 15	83 ± 16
SVC (linear)		79 ± 10	79 ± 10	82 ± 11	78 ± 12
SVC		84 ± 10	84 ± 10	87 ± 9	84 ± 10
AdaBoost classifier		66 ± 7	66 ± 7	57 ± 14	59 ± 10

[1] Feature selection method: Permutation analysis. LDA – linear discriminant analysis. SVC – support vector classification. Nested stratified k-folds cross validation: k=10 (outer loop), k=9 (inner loop)
[2] Two-sided 5x2 CV F-test p-values: full and reduced feature αSA = 0.62 (no significant difference), full αSA and dummy classifier = 2 x10^{-6} (full αSA significantly better performance)

Supplementary Table 8. αSA predictions for individual tea brands

Tea Sample	Overall accuracy (%)	Number of predictions[1]			Correct prediction[2]
		Black	Green	Grey	
Black 1	100	6	0	0	Yes
Black 2	50	3	0	3	No
Black 3	83	5	0	1	Yes
Black 4	100	6	0	0	Yes
Black 5	33	2	0	4	No
Black 6	80	4	0	1	Yes
Black 7	83	5	0	1	Yes
Black 8	100	6	0	0	Yes
Black 9	83	5	0	1	Yes
Black 10	83	5	0	1	Yes
Green 1	100	0	6	0	Yes
Green 2	100	0	6	0	Yes
Green 3	100	0	5	0	Yes
Green 4	100	0	6	0	Yes
Green 5	83	0	5	1	Yes
Green 6	100	0	6	0	Yes
Green 7	100	0	6	0	Yes
Green 8	100	0	6	0	Yes
Green 9	100	0	6	0	Yes
Green 10	100	0	6	0	Yes
Grey 1	67	2	0	4	Yes
Grey 2	83	1	0	5	Yes
Grey 3	100	0	0	6	Yes
Grey 4	67	1	1	4	Yes
Grey 5	83	1	0	5	Yes
Grey 6	100	0	0	6	Yes
Grey 7	33	4	0	2	No
Grey 8	83	0	1	5	Yes
Grey 9	100	0	0	6	Yes
Grey 10	67	2	0	4	Yes

[1] Most prominent prediction shaded the relevant colour

[2] A correct prediction occurs if most prominent prediction matches true sample label

Supplementary Table 9. Details of commercial sera samples analysed using the αSA

ID	Collection Date Range	Diagnosis	Sex	Mean Age	Ethnicity	Mean Height (cm)	Mean Weight (kg)	Mean BMI (kg m^{-2})	Medical history[1,2,3]
1	18/05/2017 – 20/03/2020	Control	F	71 ± 6	Caucasian	166 ± 4	78 ± 7	28 ± 2	DM
2	18/05/2017 – 20/03/2020	Control	F	71 ± 6	Caucasian	166 ± 4	78 ± 7	28 ± 2	H
3	18/05/2017 – 20/03/2020	Control	F	71 ± 6	Caucasian	166 ± 4	78 ± 7	28 ± 2	Hy
4	18/05/2017 – 20/03/2020	Control	F	71 ± 6	Caucasian	166 ± 4	78 ± 7	28 ± 2	H, HF
5	18/05/2017 – 20/03/2020	Control	F	71 ± 6	Caucasian	166 ± 4	78 ± 7	28 ± 2	H, O
6	18/05/2017 – 20/03/2020	Control	F	71 ± 6	Caucasian	166 ± 4	78 ± 7	28 ± 2	H
7	18/05/2017 – 20/03/2020	Control	F	71 ± 6	Caucasian	166 ± 4	78 ± 7	28 ± 2	H, VV
8	18/05/2017 – 20/03/2020	Control	F	71 ± 6	Caucasian	166 ± 4	78 ± 7	28 ± 2	H, HF
9	18/05/2017 – 20/03/2020	Control	F	71 ± 6	Caucasian	166 ± 4	78 ± 7	28 ± 2	MVS
10	18/05/2017 – 20/03/2020	Control	F	71 ± 6	Caucasian	166 ± 4	78 ± 7	28 ± 2	MVI
11	18/05/2017 – 20/03/2020	Control	F	71 ± 6	Caucasian	166 ± 4	78 ± 7	28 ± 2	O
12	18/05/2017 – 20/03/2020	Control	F	71 ± 6	Caucasian	166 ± 4	78 ± 7	28 ± 2	DM, H
13	18/05/2017 – 20/03/2020	Control	F	71 ± 6	Caucasian	166 ± 4	78 ± 7	28 ± 2	H
14	18/05/2017 – 20/03/2020	Control	F	71 ± 6	Caucasian	166 ± 4	78 ± 7	28 ± 2	A
15	23/03/2019 – 13/06/2019	NASH	F	73 ± 3	Caucasian	167 ± 3	83 ± 5	30 ± 2	CAD, H, O
16	23/03/2019 – 13/06/2019	NASH	F	73 ± 3	Caucasian	167 ± 3	83 ± 5	30 ± 2	CAD, H, O
17	23/03/2019 – 13/06/2019	NASH	F	73 ± 3	Caucasian	167 ± 3	83 ± 5	30 ± 2	CAD, H
18	23/03/2019 – 13/06/2019	NASH	F	73 ± 3	Caucasian	167 ± 3	83 ± 5	30 ± 2	CAD, H, O
19	23/03/2019 – 13/06/2019	NASH	F	73 ± 3	Caucasian	167 ± 3	83 ± 5	30 ± 2	CAD, H
20	23/03/2019 – 13/06/2019	NASH	F	73 ± 3	Caucasian	167 ± 3	83 ± 5	30 ± 2	CAD, H
21	23/03/2019 – 13/06/2019	NASH	F	73 ± 3	Caucasian	167 ± 3	83 ± 5	30 ± 2	CAD, H, O
22	23/03/2019 – 13/06/2019	NASH	F	73 ± 3	Caucasian	167 ± 3	83 ± 5	30 ± 2	CAD, H
23	23/03/2019 – 13/06/2019	NASH	F	73 ± 3	Caucasian	167 ± 3	83 ± 5	30 ± 2	CAD, H
24	23/03/2019 – 13/06/2019	NASH	F	73 ± 3	Caucasian	167 ± 3	83 ± 5	30 ± 2	CAD, H
25	23/03/2019 – 13/06/2019	NASH	F	73 ± 3	Caucasian	167 ± 3	83 ± 5	30 ± 2	CAD, H
26	23/03/2019 – 13/06/2019	NASH	F	73 ± 3	Caucasian	167 ± 3	83 ± 5	30 ± 2	CAD, H
27	23/03/2019 – 13/06/2019	NASH	F	73 ± 3	Caucasian	167 ± 3	83 ± 5	30 ± 2	CAD, H, O
28	23/03/2019 – 13/06/2019	NASH	F	73 ± 3	Caucasian	167 ± 3	83 ± 5	30 ± 2	CAD, H, O
29	09/08/2019 – 17/08/2020	CAD	F	67 ± 7	Caucasian	165 ± 5	76 ± 8	28 ± 2	O
30	09/08/2019 – 17/08/2020	CAD	F	67 ± 7	Caucasian	165 ± 5	76 ± 8	28 ± 2	H
31	09/08/2019 – 17/08/2020	CAD	F	67 ± 7	Caucasian	165 ± 5	76 ± 8	28 ± 2	nd
32	09/08/2019 – 17/08/2020	CAD	F	67 ± 7	Caucasian	165 ± 5	76 ± 8	28 ± 2	nd
33	09/08/2019 – 17/08/2020	CAD	F	67 ± 7	Caucasian	165 ± 5	76 ± 8	28 ± 2	C
34	09/08/2019 – 17/08/2020	CAD	F	67 ± 7	Caucasian	165 ± 5	76 ± 8	28 ± 2	GU, O
35	09/08/2019 – 17/08/2020	CAD	F	67 ± 7	Caucasian	165 ± 5	76 ± 8	28 ± 2	nd
36	09/08/2019 – 17/08/2020	CAD	F	67 ± 7	Caucasian	165 ± 5	76 ± 8	28 ± 2	nd
37	09/08/2019 – 17/08/2020	CAD	F	67 ± 7	Caucasian	165 ± 5	76 ± 8	28 ± 2	nd
38	09/08/2019 – 17/08/2020	CAD	F	67 ± 7	Caucasian	165 ± 5	76 ± 8	28 ± 2	nd
39	09/08/2019 – 17/08/2020	CAD	F	67 ± 7	Caucasian	165 ± 5	76 ± 8	28 ± 2	nd
40	09/08/2019 – 17/08/2020	CAD	F	67 ± 7	Caucasian	165 ± 5	76 ± 8	28 ± 2	MU, CC
41	09/08/2019 – 17/08/2020	CAD	F	67 ± 7	Caucasian	165 ± 5	76 ± 8	28 ± 2	nd
42	09/08/2019 – 17/08/2020	CAD	F	67 ± 7	Caucasian	165 ± 5	76 ± 8	28 ± 2	TN

[1] Medical history provided "as is" by the commercial biobank. No information is provided regarding being current or historic comorbidities
[2] DM – Diabetes mellitus type 2; H – Hypertension; HF – Heart failure; O – Obesity; VV – Varicose veins; MVS – Mitral valve stenosis; MVI – Mitral valve insufficiency; A – Anaemia; C – Cholelithiasis; GU – Gastric ulcer; MU – Myoma of uterus; CC – Chronic cholecystitis; TN – Thyroid node
[3] nd – No medical history information was provided with sample

Supplementary Table 10. Model parameters and results for the classification of NASH and non-NASH samples by the αSA

Algorithm[1]	Features[2]	Accuracy (%)	Recall (%)	Precision (%)	F1 score (%)
Dummy classifier (popular)	5	66 ± 5	66 ± 5	44 ± 7	52 ± 7
Dummy classifier (stratified)	5	78 ± 5	78 ± 5	84 ± 3	74 ± 7
K-neighbors classifier	5	88 ± 18	88 ± 18	89 ± 16	88 ± 17
GaussianNB	5	85 ± 11	85 ± 11	86 ± 11	85 ± 11
Linear Discriminant analysis	5	90 ± 5	90 ± 5	93 ± 4	90 ± 6
SVC (linear)	5	90 ± 5	90 ± 5	93 ± 4	90 ± 6
SVC (rbf)	5	83 ± 7	83 ± 7	87 ± 7	83 ± 7
AdaBoost classifier	5	85 ± 11	85 ± 11	86 ± 11	85 ± 11

[1] Feature selection method: Permutation analysis. LDA – linear discriminant analysis. SVC – support vector classification. No class balancing applied. Nested stratified k-folds cross validation: k=5 (inner and outer loops)
[2] Two-sided 5x2 CV F-test p-values: full and reduced feature αSA = 0.46 (no significant difference), full αSA and dummy classifier (stratified) = 0.038 (full αSA significantly better performance), full αSA and dummy classifier (popular) = 0.003 (full αSA significantly better performance)

Supplementary Table 11. Model parameters and results for the classification of NASH, CAD and control samples by the αSA

Algorithm[1]	Features[2]	Accuracy (%)	Recall (%)	Precision (%)	F1 score (%)
Dummy classifier (popular)	4	28 ± 5	28 ± 5	8 ± 3	13 ± 4
Dummy classifier (stratified)	4	36 ± 19	36 ± 19	23 ± 16	28 ± 17
K-neighbors classifier	4	69 ± 12	69 ± 12	76 ± 11	68 ± 12
GaussianNB	4	74 ± 17	74 ± 17	74 ± 21	71 ± 20
Linear Discriminant analysis	4	74 ± 15	74 ± 15	80 ± 11	74 ± 14
SVC (linear)	4	64 ± 22	64 ± 22	67 ± 22	64 ± 23
SVC (rbf)	4	67 ± 20	67 ± 20	70 ± 24	65 ± 21
AdaBoost classifier	4	54 ± 13	54 ± 13	52 ± 22	50 ± 16

[1] Feature selection method: Permutation analysis. LDA – linear discriminant analysis. SVC – support vector classification. Nested stratified k-folds cross validation: k=5 (inner and outer loops)
[2] Two-sided 5x2 CV F-test p-values: full and reduced feature αSA = 0.46 (no significant difference), full αSA and dummy classifier = 0.004 (full αSA significantly better performance)

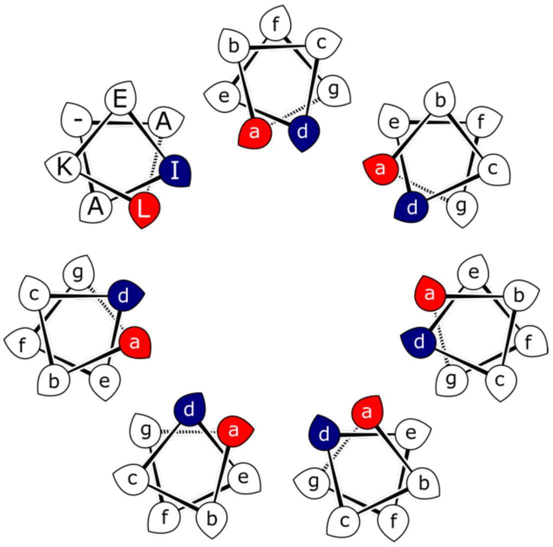

Supplementary Figure 1. Helical wheel of an αHB. The *a* and *d* sites of the heptad sequence repeat, *abcdefg*, are highlighted in red and blue, respectively. The sequence of CC-Type2-[L_aI_d]$_4$ (peptide ID 3) is shown as an example in one of the helices.

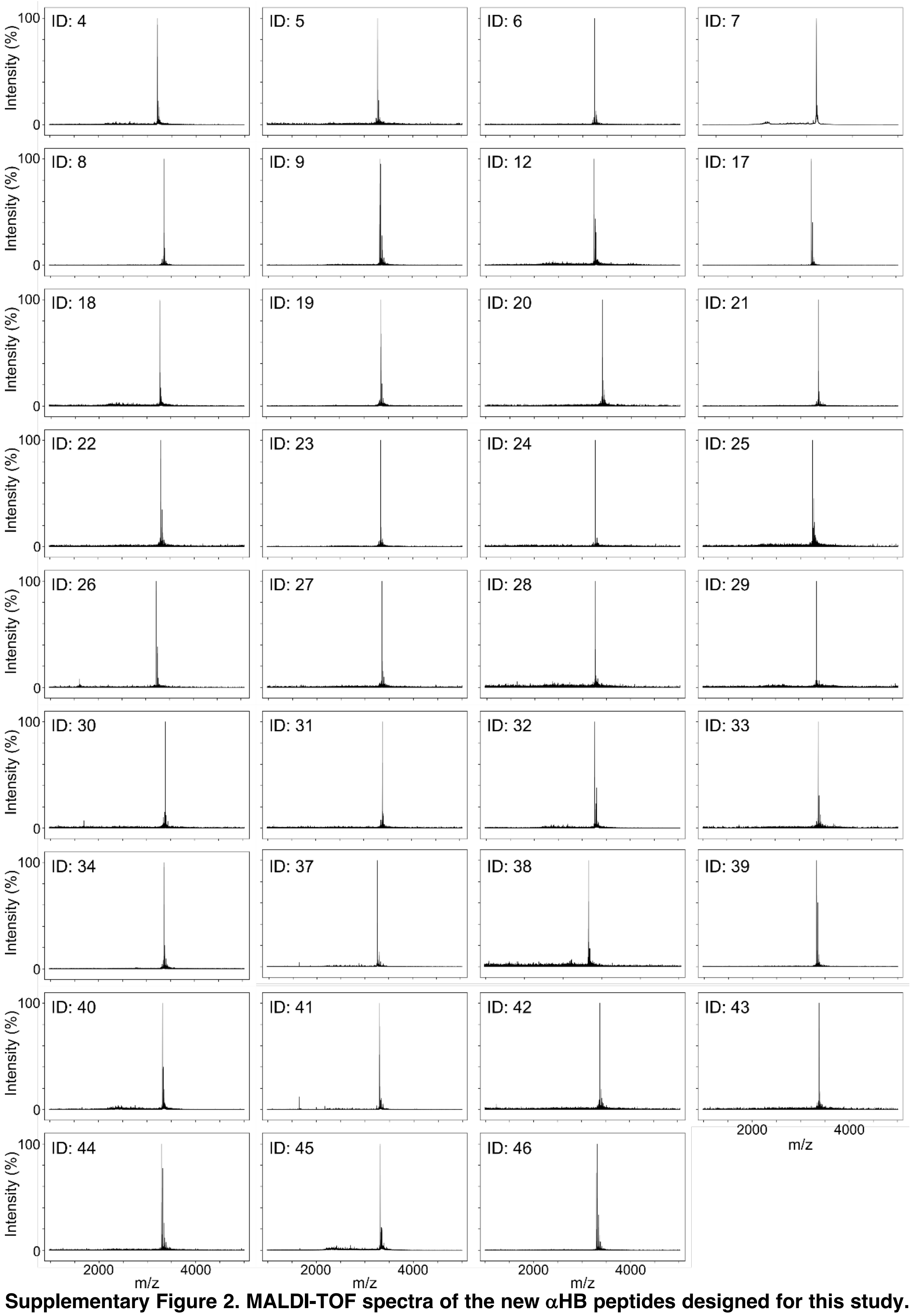

Supplementary Figure 2. MALDI-TOF spectra of the new αHB peptides designed for this study. Sequences, calculated mass and observed mass for individual peptide IDs can be found in Supplementary Table 1. Source data are provided as a Source Data file.

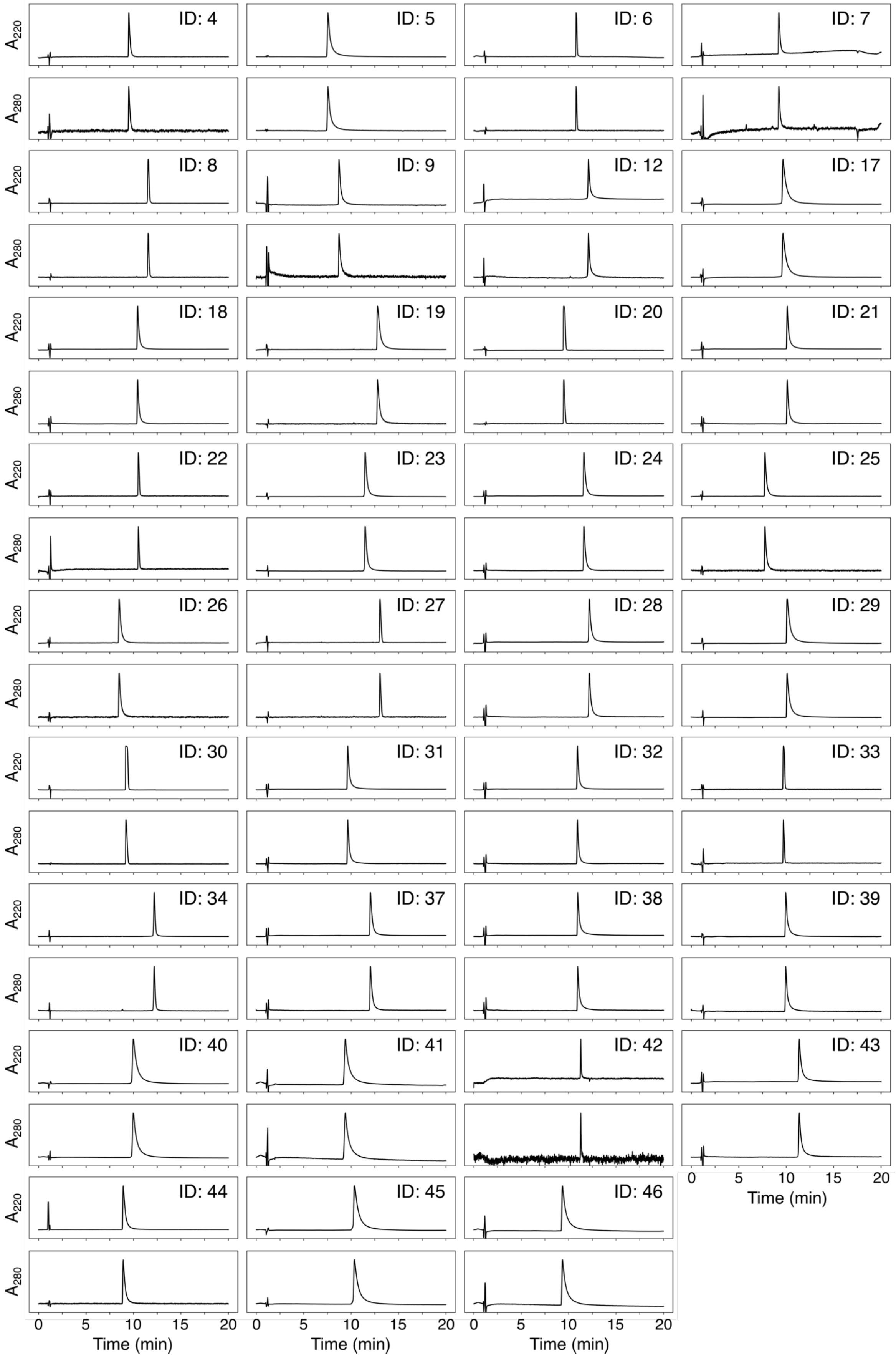

Supplementary Figure 3. Analytical HPLC traces of the new αHB peptides designed for this study. Top: Analytical HPLC chromatogram at 220 nm. Bottom: Analytical HPLC chromatogram at 280

nm. Sequences for individual peptides can be found in Supplementary Table 1. Source data are provided as a Source Data file.

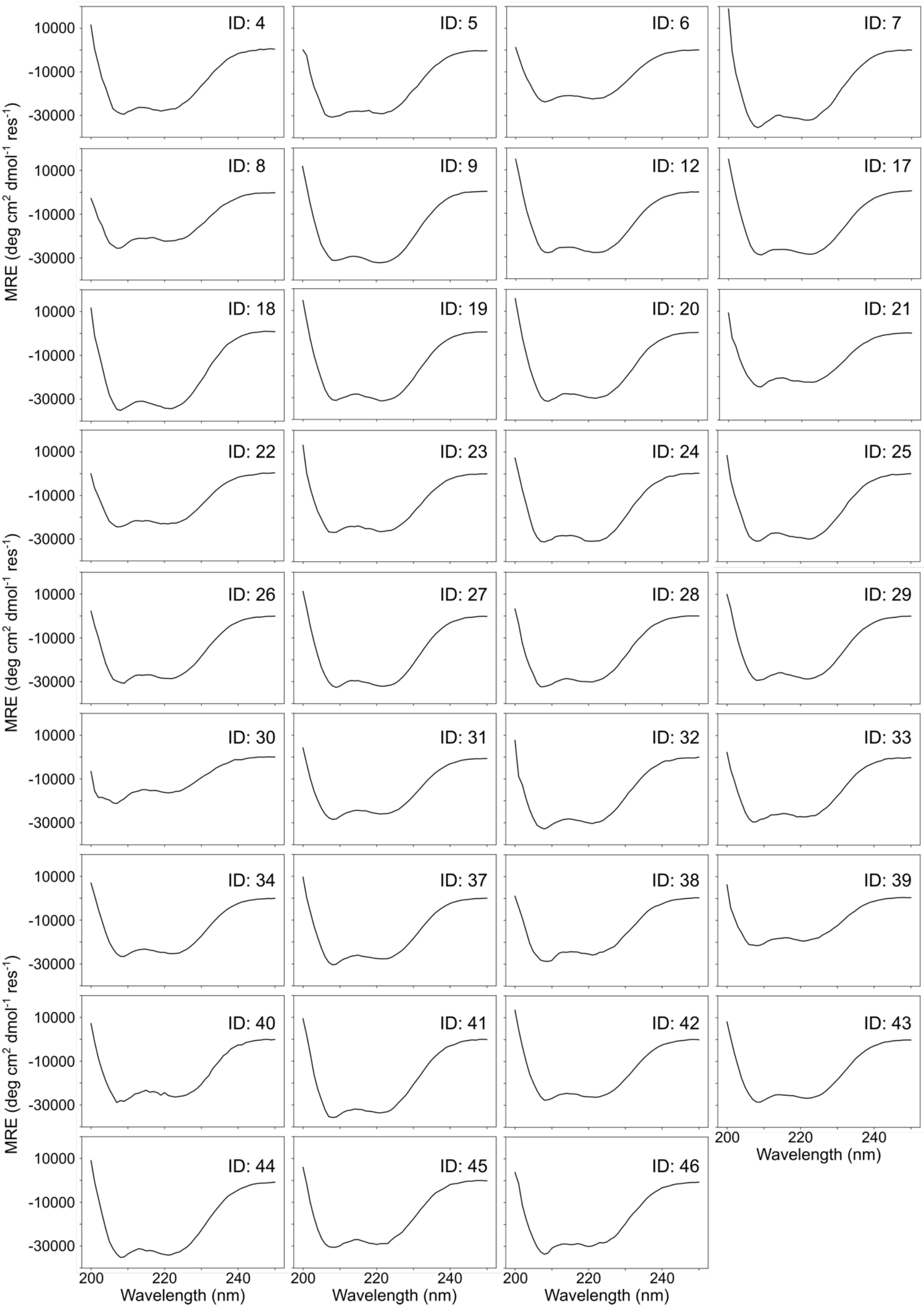

Supplementary Figure 4. CD spectra of the new αHB peptides designed for this study. Sequences for individual peptides numbered can be found in Supplementary Table 1. Conditions: 10 μM peptide, PBS, pH 7.4, 20 °C. Source data are provided as a Source Data file.

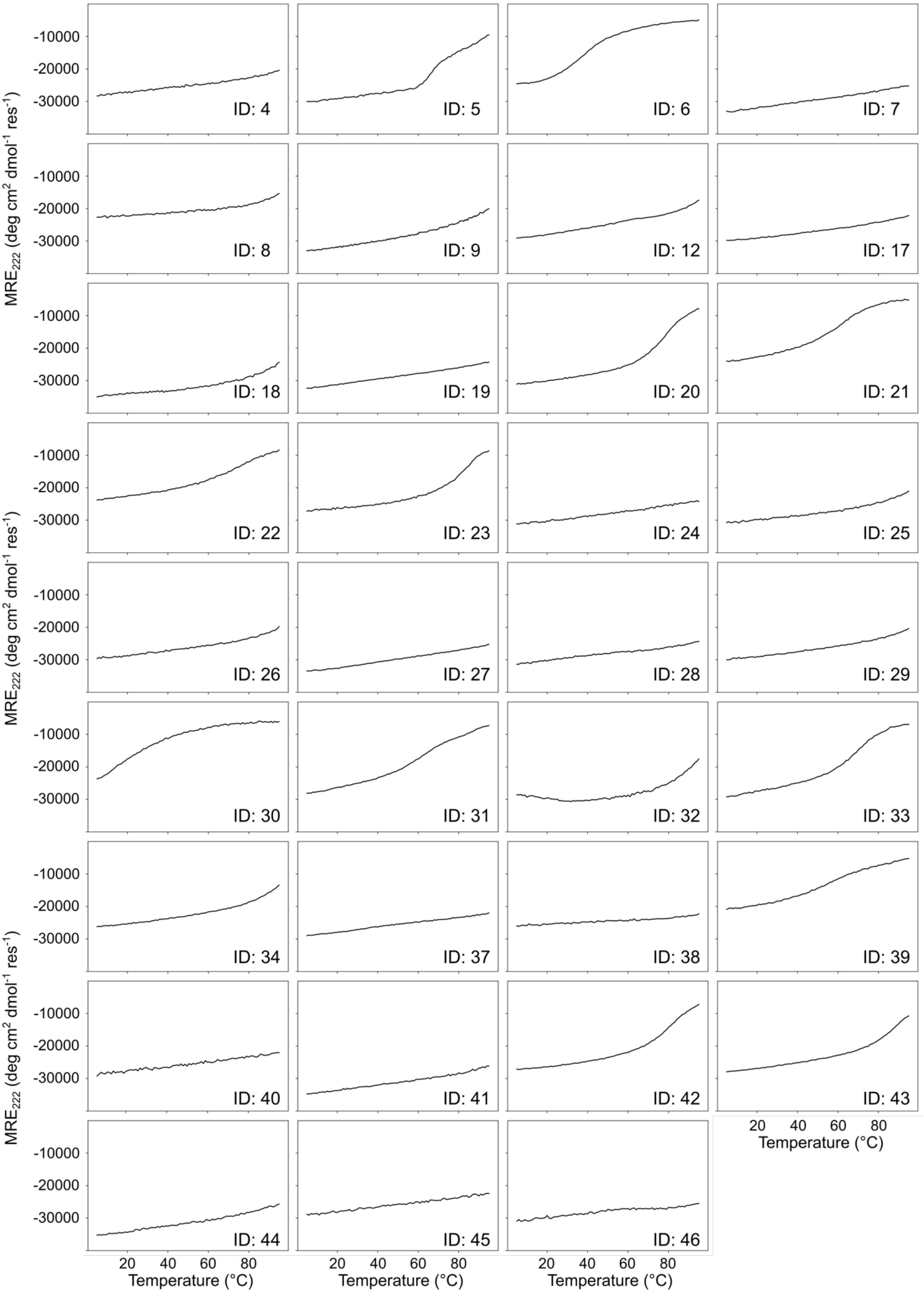

Supplementary Figure 5. CD thermal denaturation profiles of the new αHB peptides designed for this study. Sequences for individual peptides can be found in Supplementary Table 1. Conditions: 10 μM peptide, PBS, pH 7.4, 5-95 °C. Source data are provided as a Source Data file.

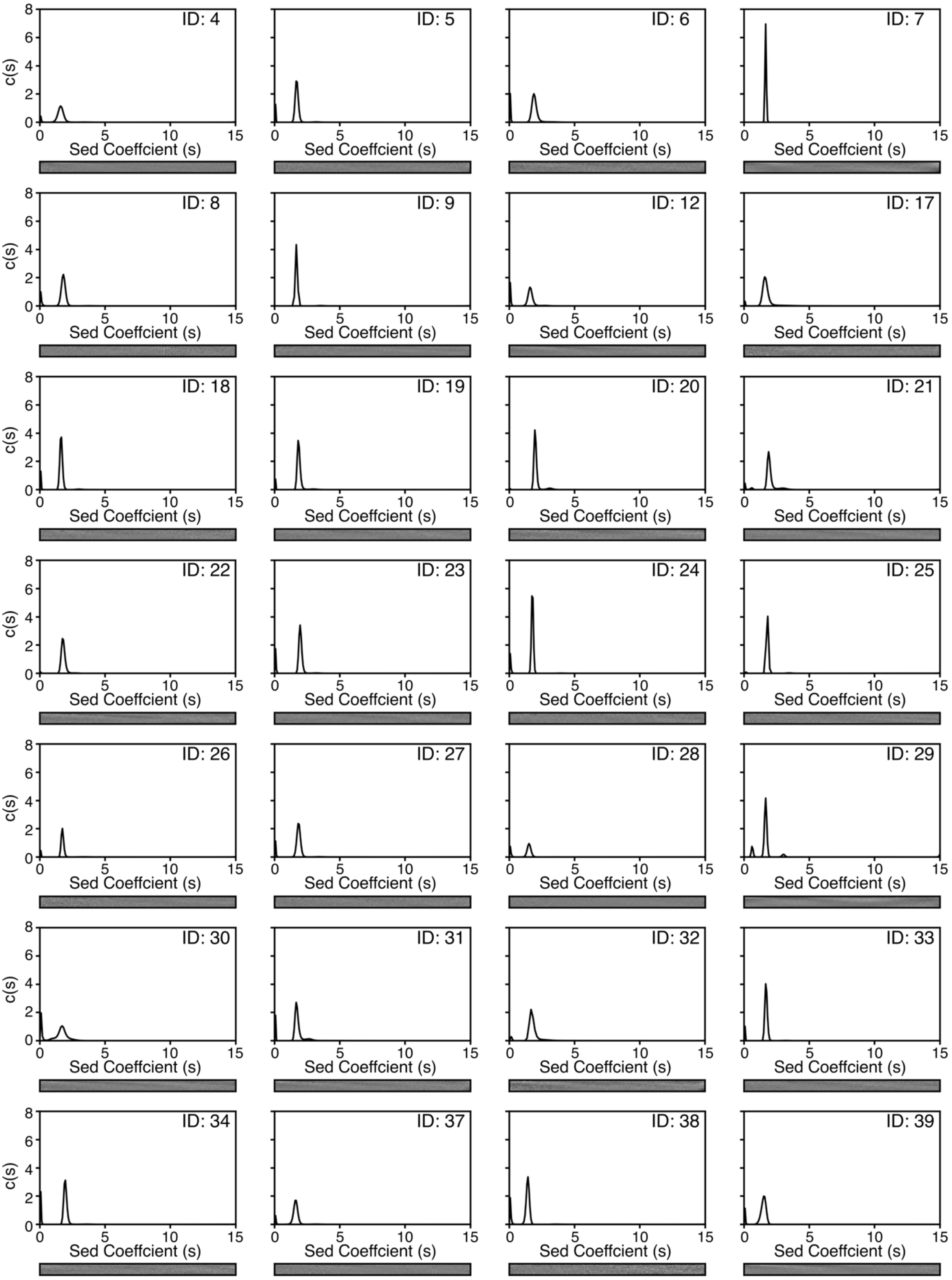

Supplementary Figure 6. Sedimentation velocity (SV) AUC traces of the new αHB peptides designed for this study. Sequences for individual peptides can be found in Supplementary Table 1. Fit data for individual peptides can be found in Supplementary Table 2. Residuals are shown as a bitmap below the fitted data. Conditions: 150 μM peptide, PBS, pH 7.4, 20 °C. Source data are provided as a Source Data file.

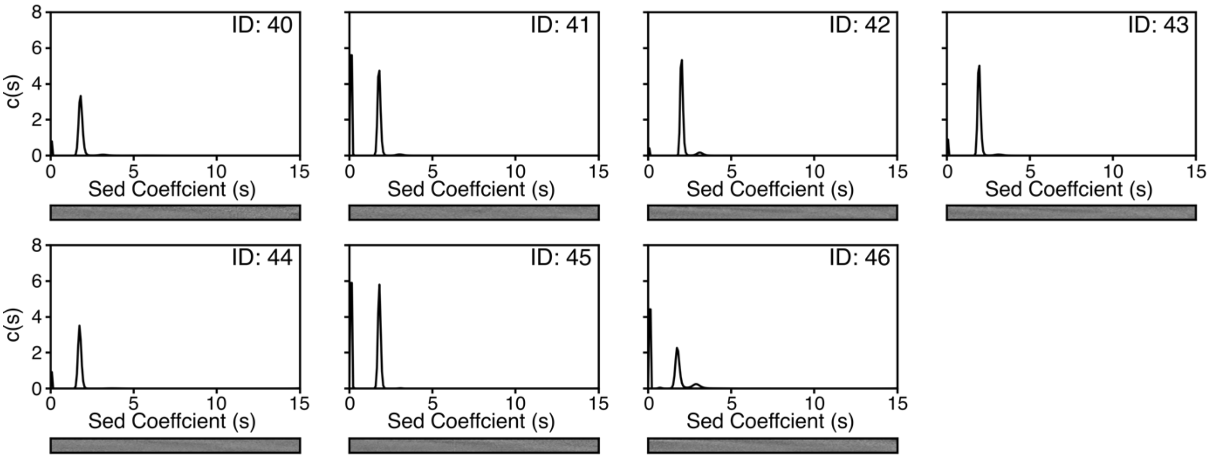

Supplementary Figure 6 continued. Sedimentation velocity (SV) AUC traces of the new αHB peptides designed for this study. Sequences for individual peptides can be found in Supplementary Table 1. Fit data for individual peptides can be found in Supplementary Table 2. Residuals are shown as a bitmap below the fitted data. Conditions: 150 μM peptide, PBS, pH 7.4, 20 °C. Source data are provided as a Source Data file.

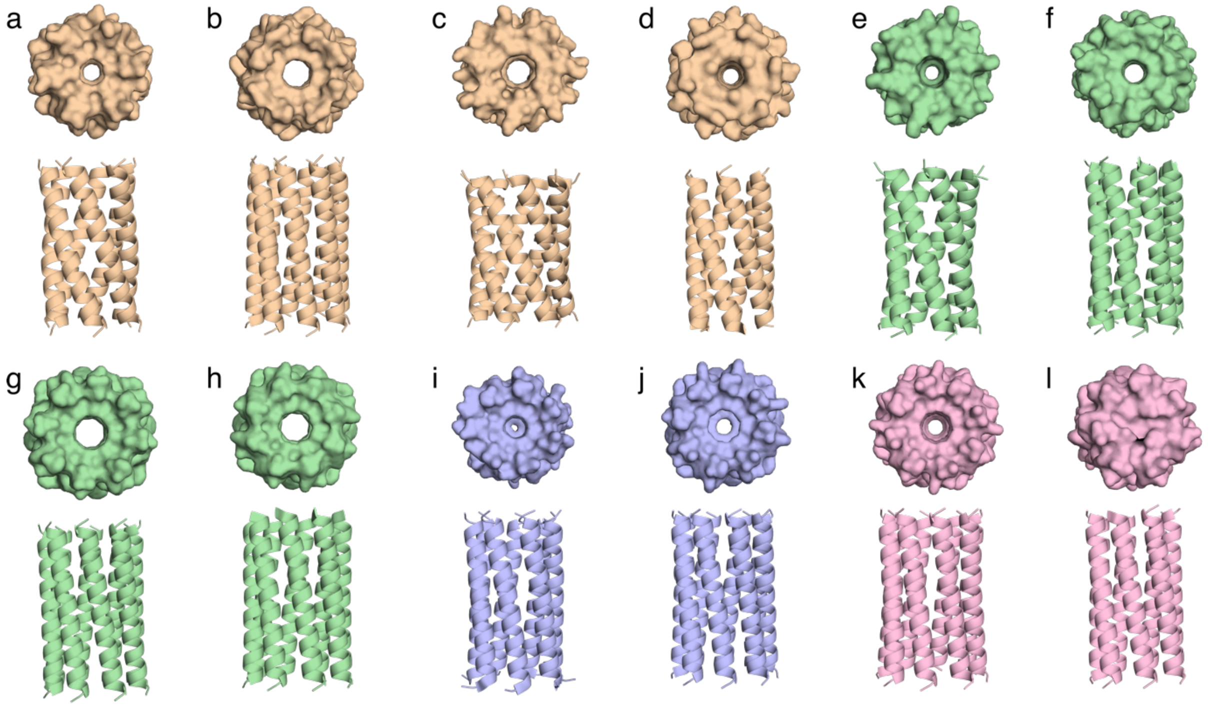

Supplementary Figure 7. Orthogonal views of new X-ray crystal structures of αHB peptides determined through this study. a, CC-Type2-[L_aI_d]$_4$-L14A, ID: 4, PDB: 7NFG. **b,** CC-Type2-[L_aI_d]$_4$-I24A, ID: 7, PDB: 7NFF. **c,** CC-Type2-[M_aI_d]$_4$, ID: 9, PDB: 7NFH. **d,** CC-Type2-[$Q_gL_aI_d$]$_4$, ID: 15, PDB: 8A09. **e,** CC-Type2-[L_aI_d]$_4$-I17C, ID: 17, PDB: 7NFO. **f,** CC-Type2-[L_aI_d]$_4$-L21N-I24N, ID: 21, PDB: 7NFN. **g,** CC-Type2-[L_aI_d]$_4$-I24N, ID: 25, PDB: 7NFL. **h,** CC-Type2-[L_aI_d]$_4$-I24S, ID: 26, PDB: 7NFK. **i,** CC-Type2-[L_aI_d]$_4$-I17K, ID: 29, PDB: 7NFP. **j,** CC-Type2-[L_aI_d]$_4$-L21K, ID: 32, PDB: 7NFM. **k,** CC-Type2-[L_aI_d]$_4$-L7Y, ID: 41, PDB: 7NFI. **l,** CC-Type2-[L_aI_d]$_4$-L28Y, ID: 46, PDB: 7NFJ.

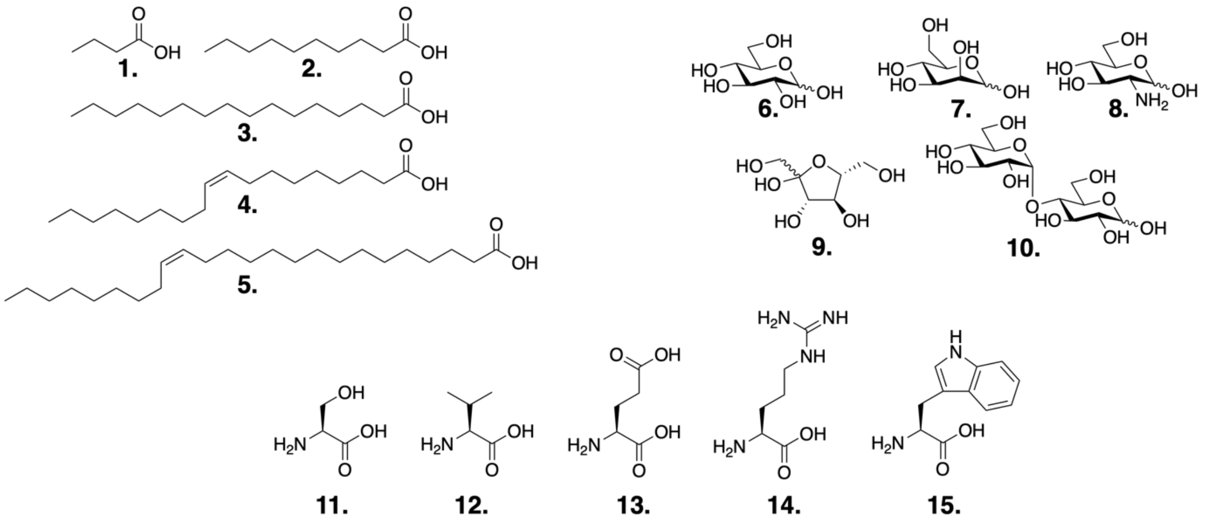

Supplementary Figure 8. Chemical structures of the fatty acids (FAs), carbohydrates (CHOs) and amino acids (AAs) analysed with the αSA. 1. Butanoic acid; 2. Decanoic acid; 3. Palmitic acid; 4. Oleic acid; 5. Nervonic acid; 6. Glucose; 7. Mannose; 8. Glucosamine; 9. Fructose; 10. Maltose; 11. Serine; 12. Valine; 13. Glutamic acid; 14. Arginine; 15. Tryptophan

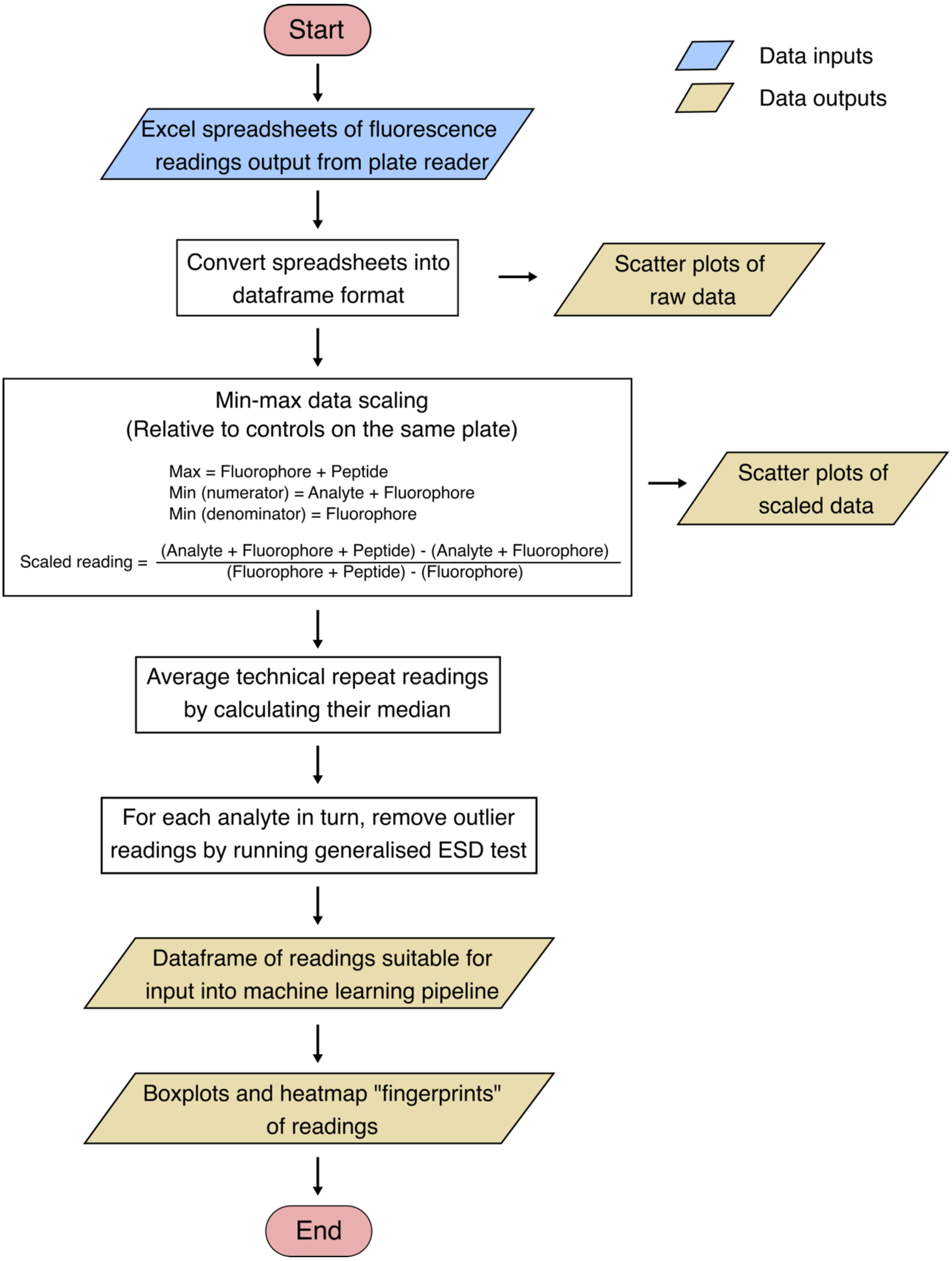

Supplementary Figure 9. The pre-processing pipeline of the αSA analysis.

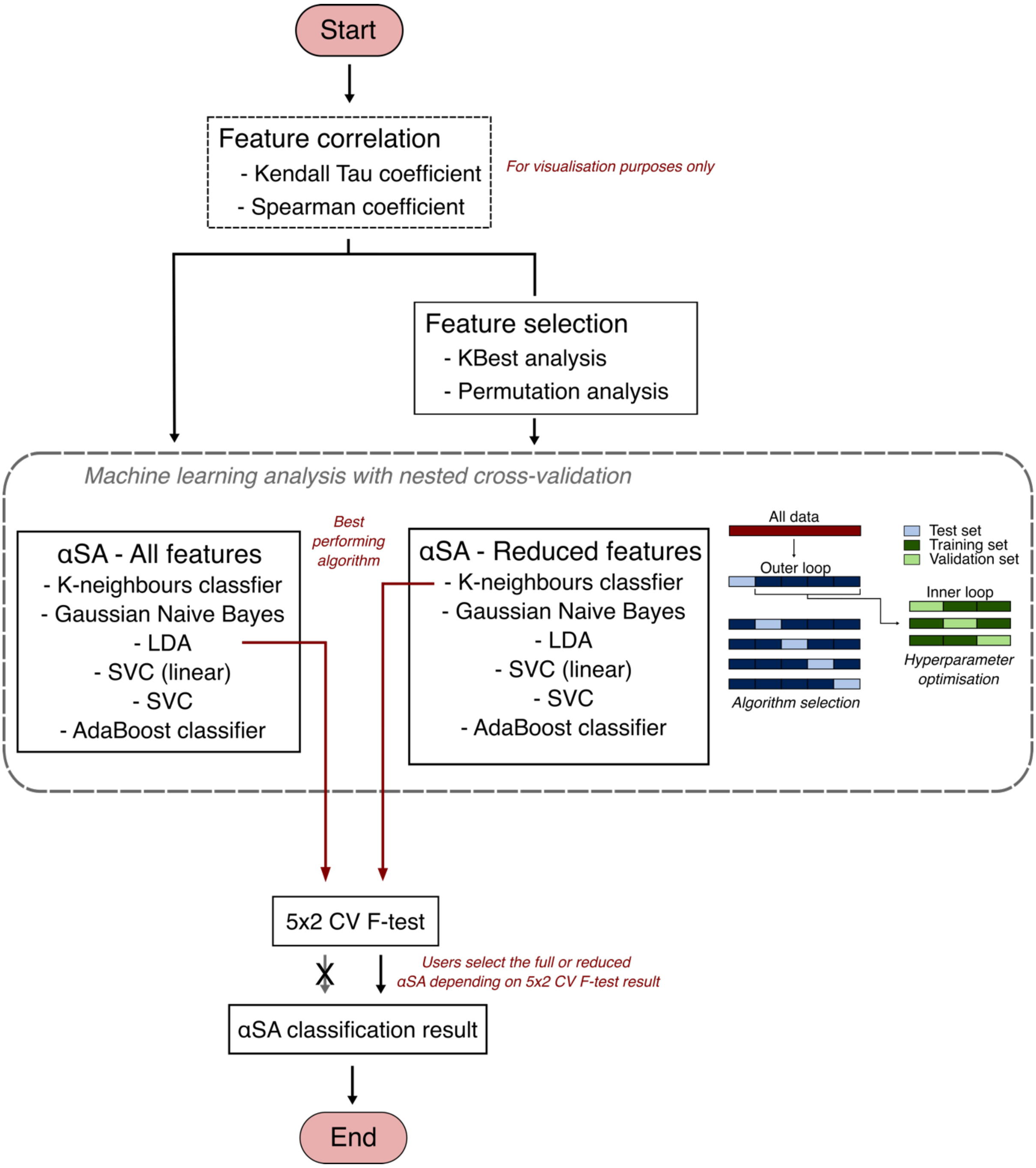

Supplementary Figure 10. The machine learning pipeline of the αSA analysis.

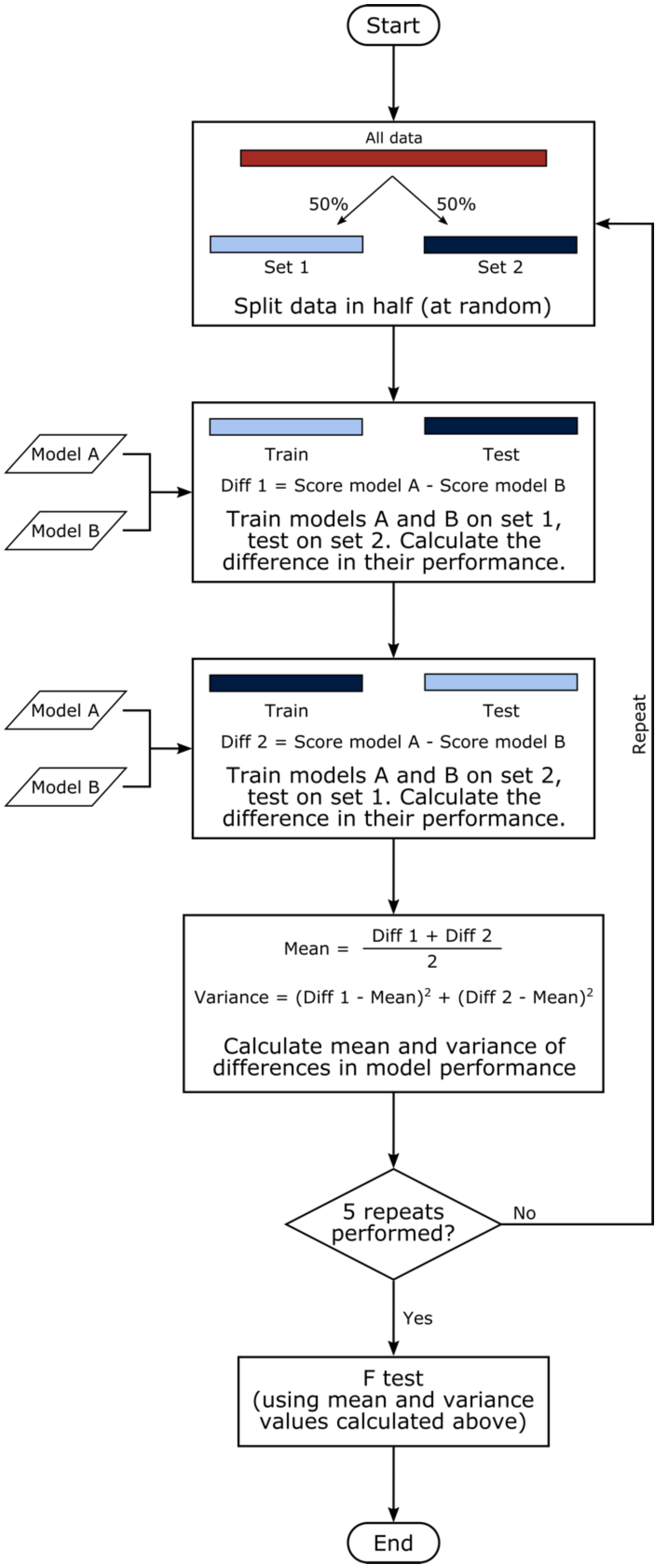

Supplementary Figure 11. Flow chart illustrating how a 5x2 CV F-test is performed.

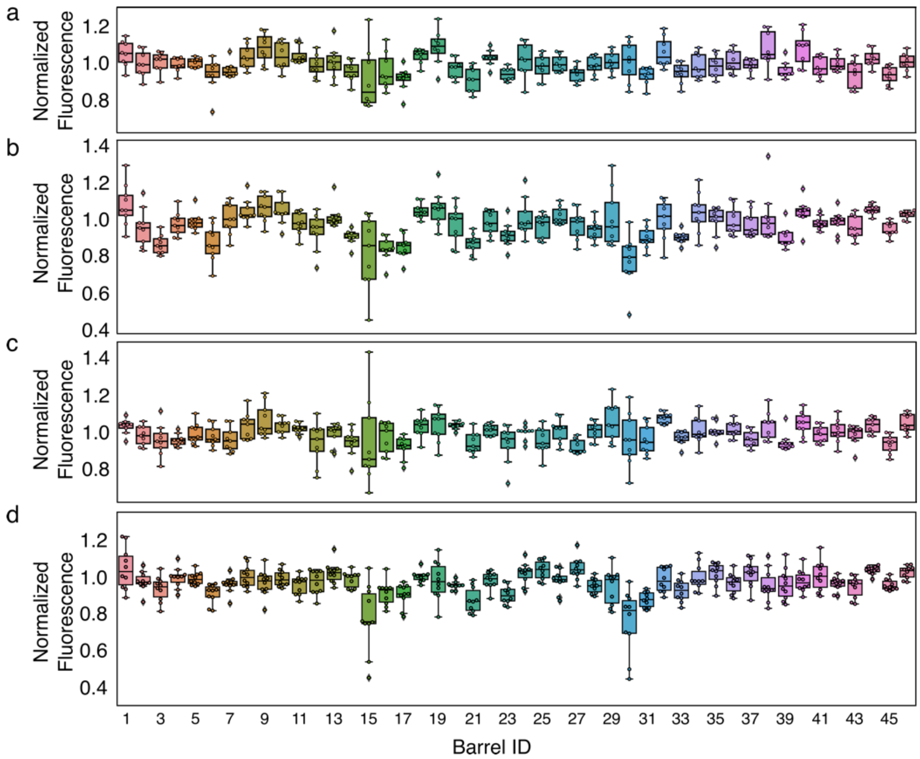

Supplementary Figure 12. Min-max scaled fluorescent signals from the αSA upon being challenged with amino acids. a, Glutamate, n=8 independent samples. **b**, Arginine, n=9 independent samples. **c**, Serine, n=9 independent samples. **d**, Valine, n=10 independent samples. **a-d**, Boxes show the interquartile range with the median presented as a line. Whiskers show 1.5 x interquartile range, or the range if a smaller value. Outliers are shown as diamonds. Source data are provided as a Source Data file.

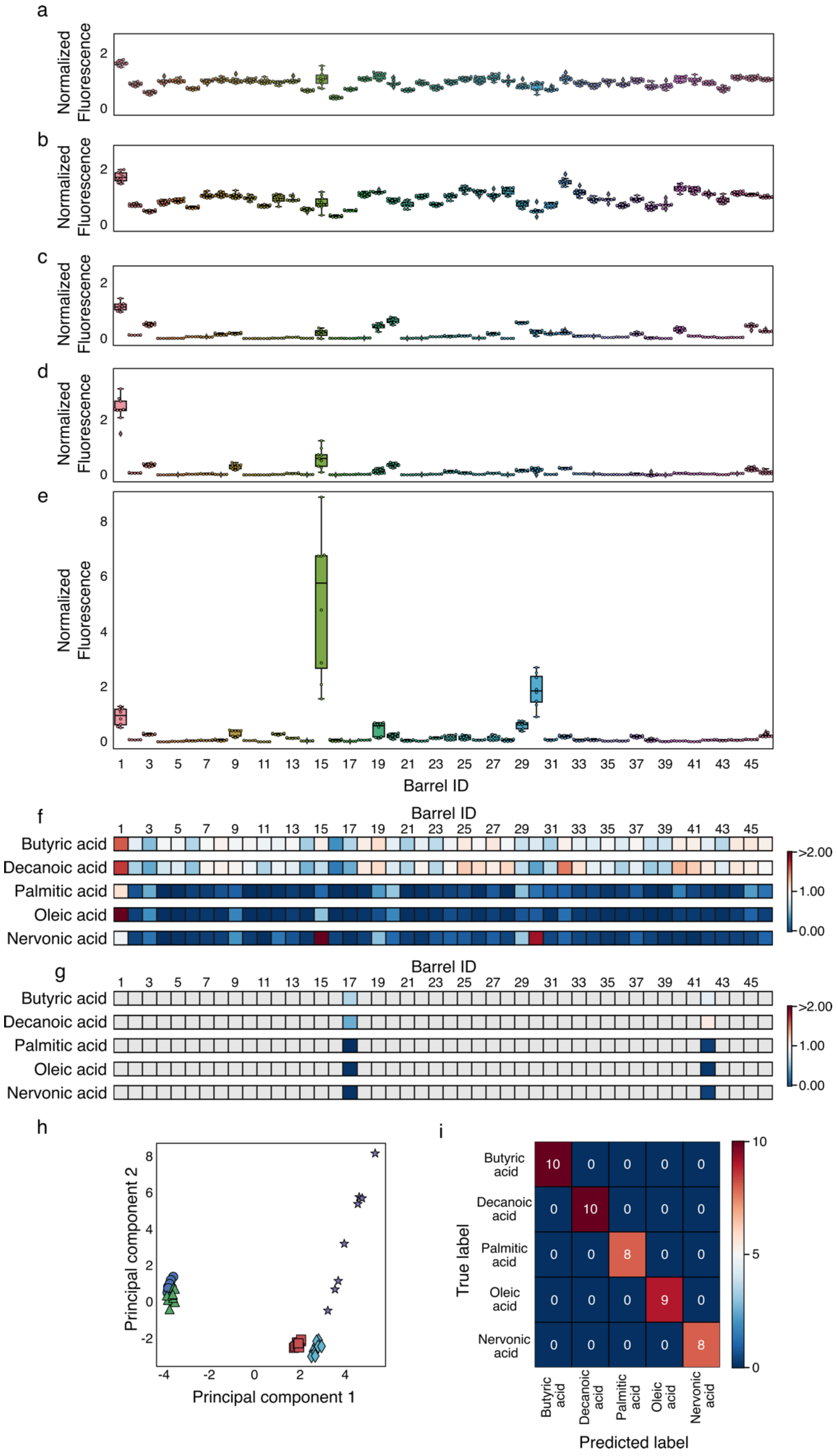

Supplementary Figure 13. αSA analysis for the differentiation of fatty acids. a-e, Min-max scaled fluorescent signals from the αSA challenged with fatty acids. Butyric acid (4:0, **a,** n=10 independent

samples), decanoic acid (10:0, **b**, n=10 independent samples), palmitic acid (16:0, **c**, n=8 independent samples), oleic acid (18:1, **d**, n=9 independent samples) and nervonic acid (24:1, **e**, n=8 independent samples). Boxes show the interquartile range with the median presented as a line. Whiskers show 1.5 x interquartile range, or the range if a smaller value. Outliers are shown as diamonds. **f,** Representative dye-displacement data for each analyte in the FA class. αHB ID is shown above each fingerprint. In these cases, min-max scaled dye displacement is colored from dark red (less displacement) to dark blue (more displacement) according to the respective heat maps (right-hand side of each panel). Each fingerprint corresponds to the median signal across all repeats for each FA. **g,** The 2 features selected to take forward to classification. Color scheme as in **f**, αHBs not selected are colored grey. **f** & **g**, Values have been limited to a maximum of 2.00 for visualization purposes only, the full range of data can be seen in panels a-e. **h**, Principal component analysis of the 5 fatty acids. Butyric acid – blue circle; decanoic acid – green triangle; palmitic acid – red square; oleic acid – cyan diamond; nervonic acid – purple star. **i**, Confusion matrices generated from predictions of FAs using 2 features (**g**) and the Gaussian Naïve Bayes algorithm with nested cross-validation. Here the coloring scheme is from dark red (all prediction) to dark blue (no predictions) according to the heat map (right-hand side). Source data are provided as a Source Data file.

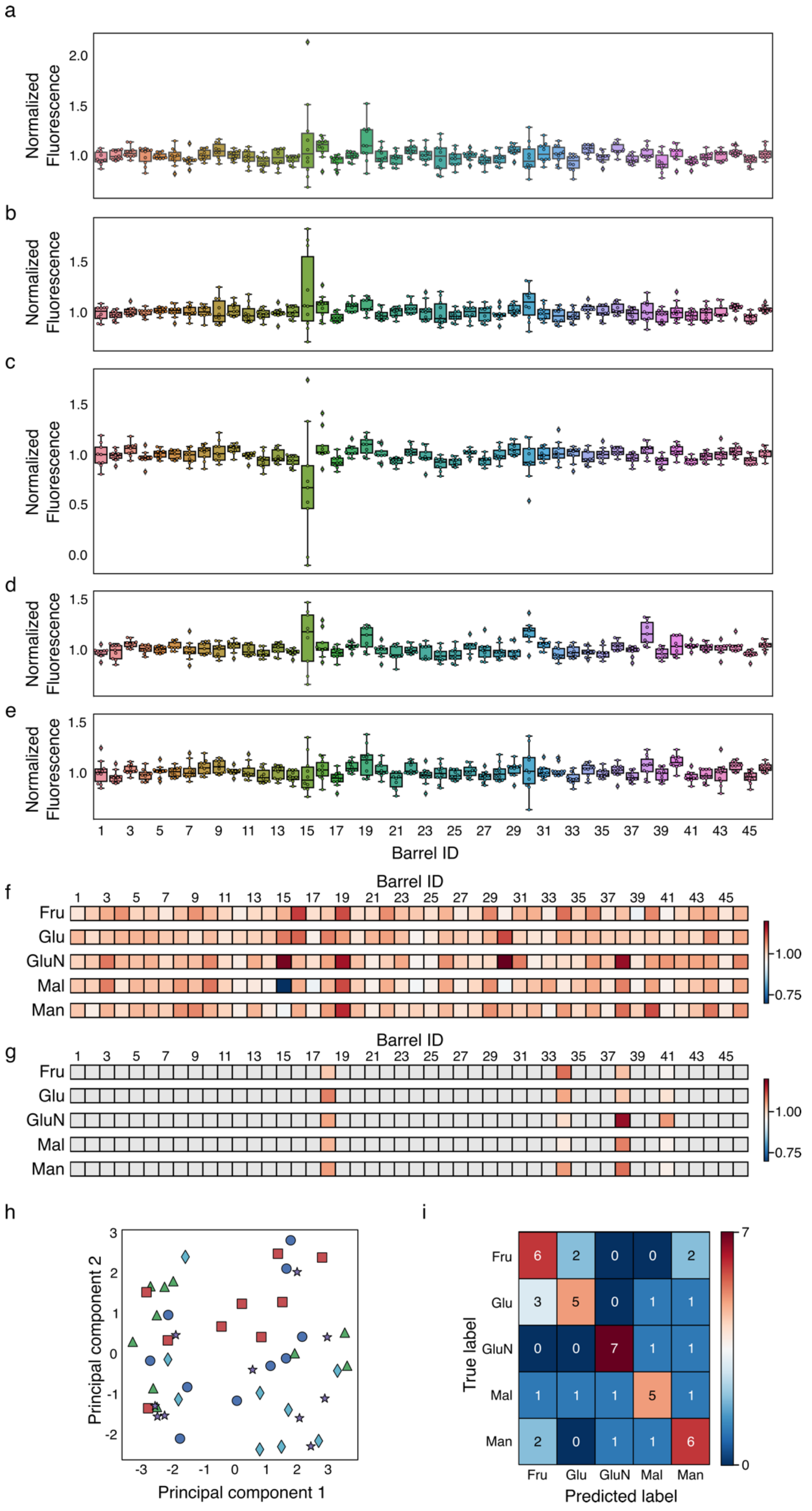

Supplementary Figure 14. αSA analysis for the differentiation of carbohydrates. a-e, Min-max scaled fluorescent signals from the αSA challenged with carbohydrates. Fructose (**a**, n=10 independent

samples), glucose (**b**, n=10 independent samples), glucosamine (**c**, n=9 independent samples), maltose (**d**, n=9 independent samples) and mannose (**e**, n=10 independent samples). Boxes show the interquartile range with the median presented as a line. Whiskers show 1.5 x interquartile range, or the range if a smaller value. Outliers are shown as diamonds. **f,** Representative dye-displacement data for each analyte in the CHO class. αHB ID is shown above each fingerprint. In these cases, min-max scaled dye displacement is colored from dark red (less displacement) to dark blue (more displacement) according to the respective heat maps (right-hand side of each panel). Each fingerprint corresponds to the median signal across all repeats for each CHO. **g,** The 4 features selected to take forward to classification. Color scheme as in **f**, αHBs not selected are colored grey. **h**, Principal component analysis of the 5 carbohydrates. Fructose – blue circle; glucose – green triangle; glucosamine – red square; maltose – cyan diamond; mannose – purple star. **i**, Confusion matrices generated from predictions of CHOs using 4 features (**g**) and the SVC algorithm with nested cross-validation. Here the coloring scheme is from dark red (all prediction) to dark blue (no predictions) according to the heat map (right-hand side). Source data are provided as a Source Data file.

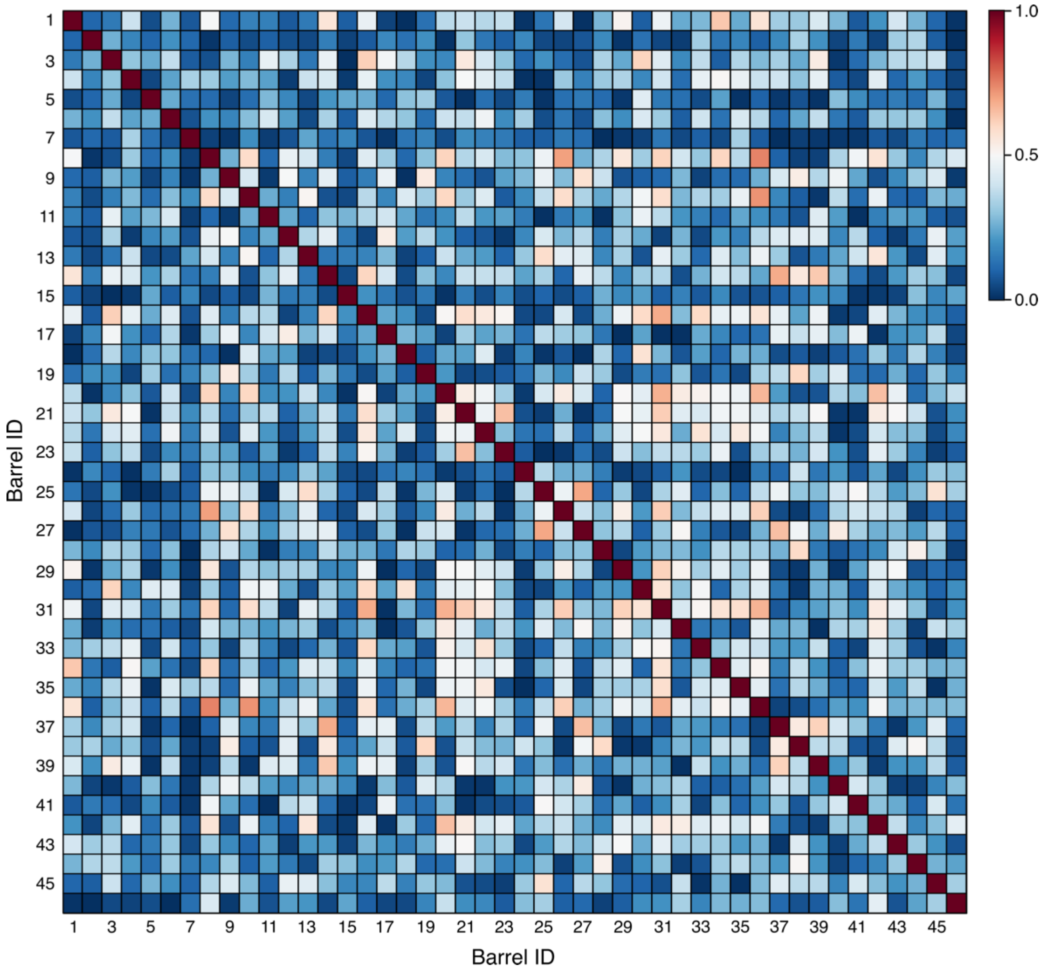

Supplementary Figure 15. Spearman coefficients of the αHBs in the αSA for the amino acid fingerprints. Color scheme is from strong correlation (dark red) to no correlation (dark blue) according to the heat map (right-hand side). Source data are provided as a Source Data file.

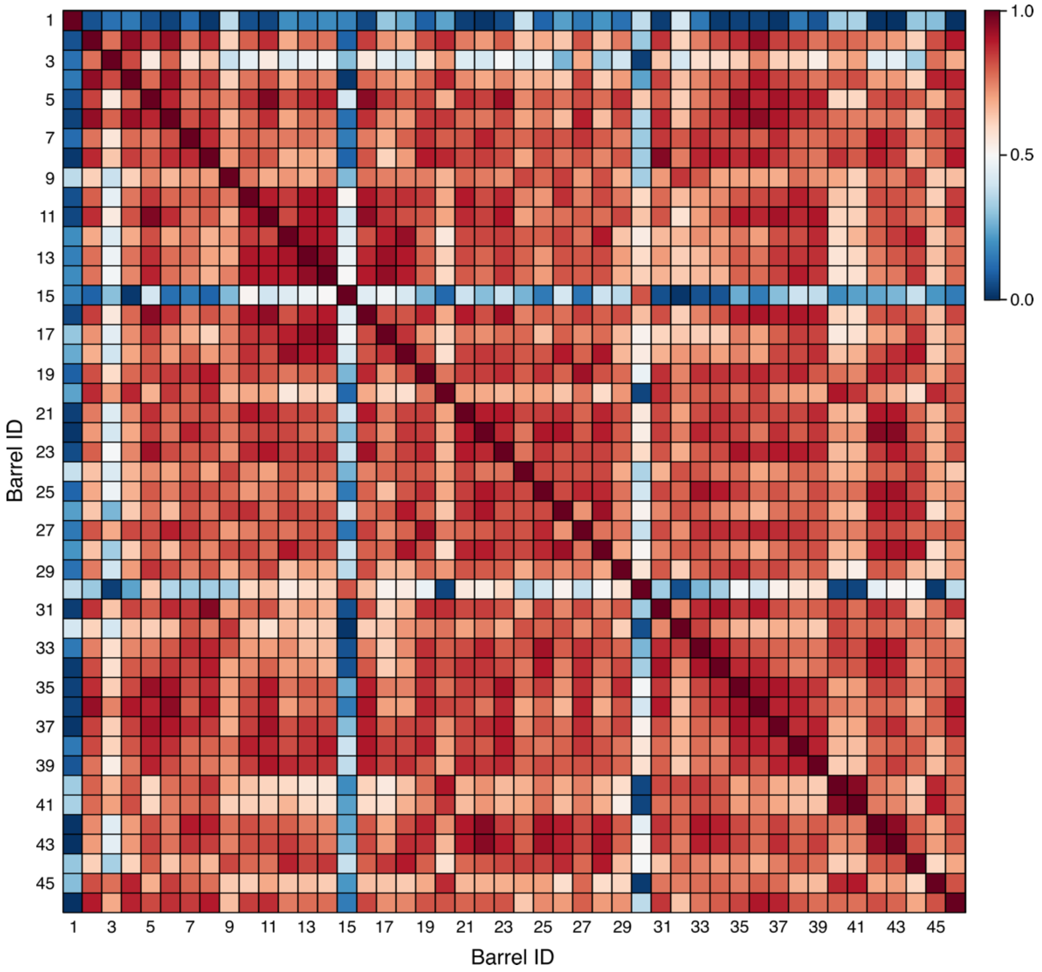

Supplementary Figure 16. Spearman coefficients of the αHBs in the αSA for the fatty acid fingerprints. Color scheme is from strong correlation (dark red) to no correlation (dark blue) according to the heat map (right-hand side). Source data are provided as a Source Data file.

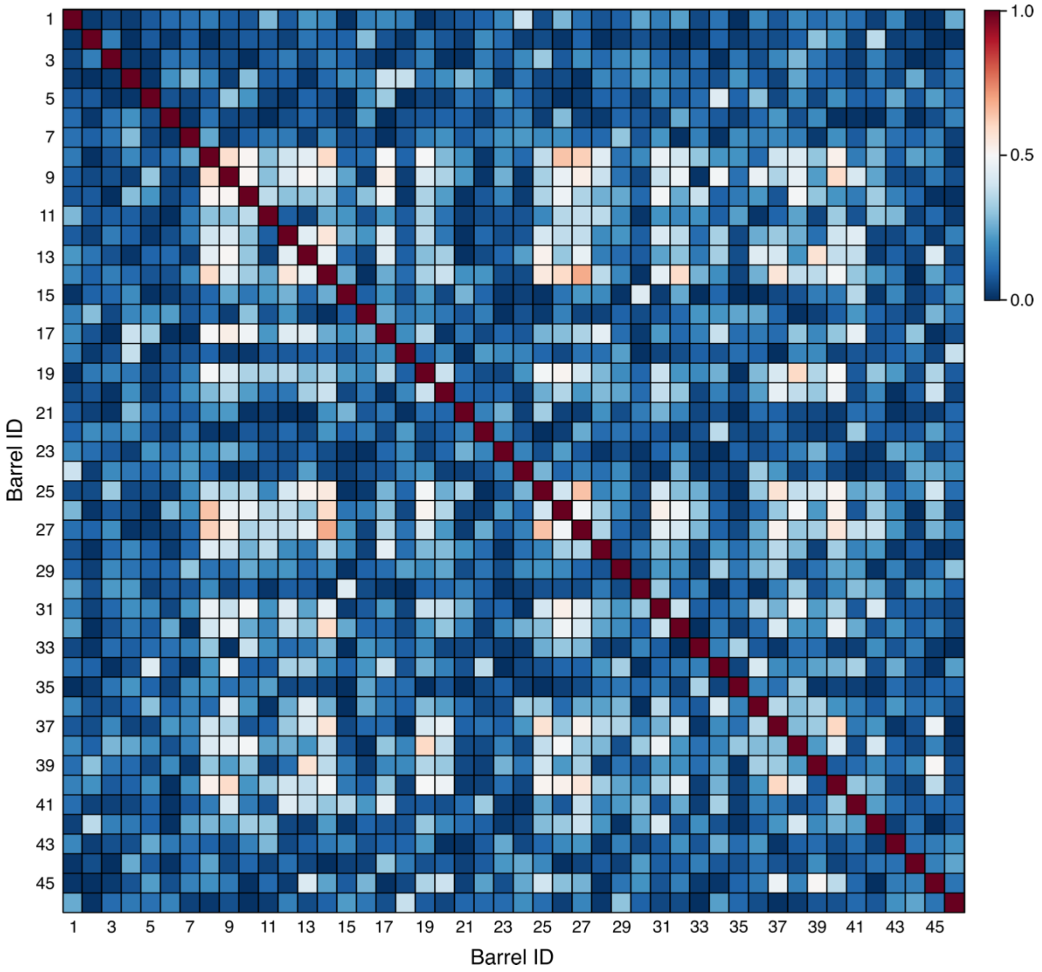

Supplementary Figure 17. Spearman coefficients of the αHBs in the αSA for the carbohydrate fingerprints. Color scheme is from strong correlation (dark red) to no correlation (dark blue) according to the heat map (right-hand side). Source data are provided as a Source Data file.

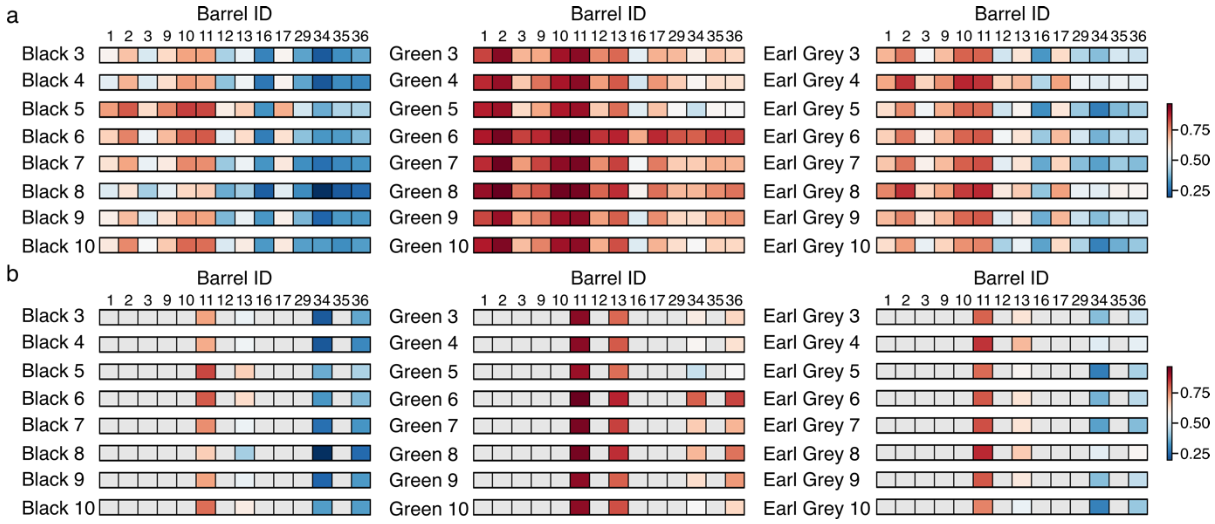

Supplementary Figure 18. Representative dye-displacement data for each tea brand. a, Representative dye-displacement data for each brand in the tea class. αHB ID is shown above each fingerprint. In these cases, min-max scaled dye displacement is colored from dark red (less displacement) to dark blue (more displacement) according to the respective heat maps (right-hand side of each panel). Each fingerprint corresponds to the median signal across all repeats for each brand of tea. **b,** The 4 features selected to take forward to classification. Color scheme as in **a**, αHBs not selected are colored grey. For visualization purposes, the fingerprints (**a** & **b**) are the median from the 6 independent repeats for each tea brand rather than the 180 individual fingerprints used in the analysis. All 180 fingerprints can be found at https://github.com/woolfson-group/array_sensing_data_analysis. The corresponding brand names can be found in Supplementary Table 8. Source data are provided as a Source Data file.

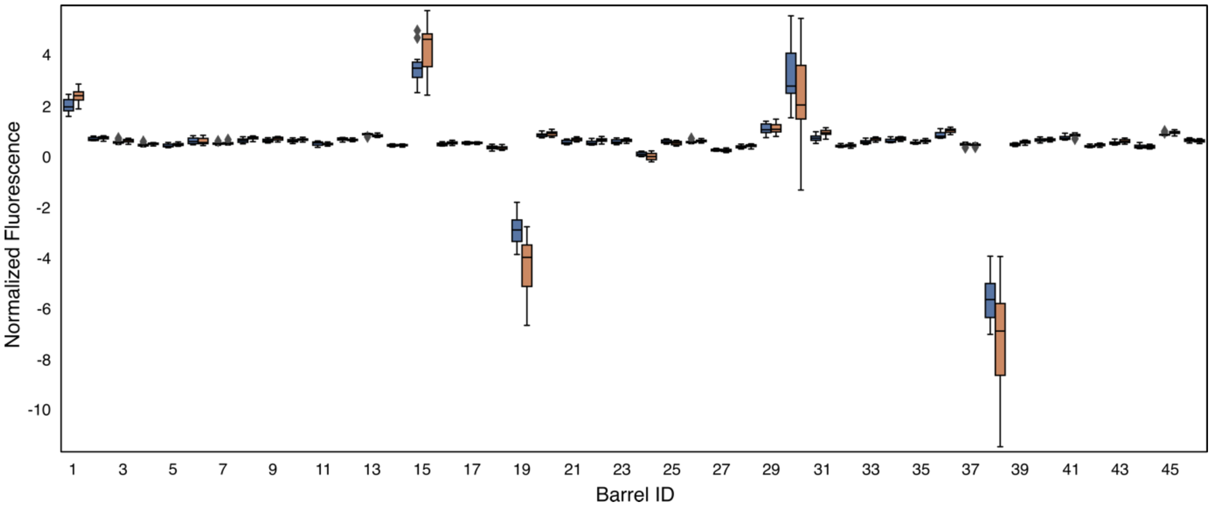

Supplementary Figure 19. Min-max scaled fluorescent signals from the αSA challenged with NASH sera samples. NASH (blue), non-NASH (orange), n=41 independent samples each measured 4 times. Boxes show the interquartile range with the median presented as a line. Whiskers show 1.5 x interquartile range, or the range if a smaller value. Outliers are shown as diamonds. Source data are provided as a Source Data file.

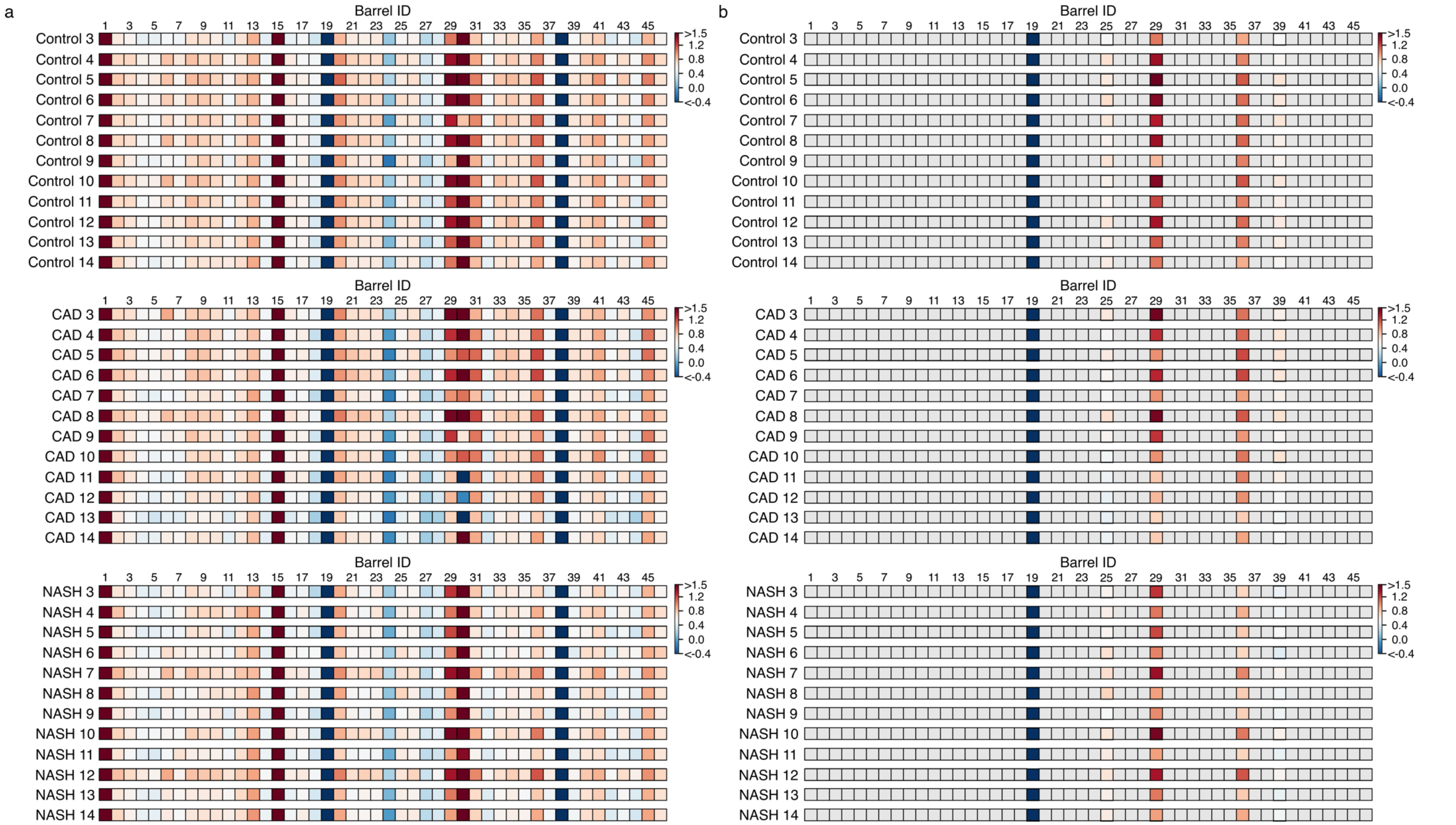

Supplementary Figure 20. Median dye-displacement data for each sera sample. The complete fingerprint (**a**) and the fingerprint of the most important features (**b**) are shown. αHB ID is shown above each fingerprint. In these cases, min-max scaled dye displacement is colored from dark red (less displacement) to dark blue (more displacement) according to the respective heat maps (right-hand side of each panel). Each fingerprint is the median value from 16 repeats

of each serum sample (4 independent repeats each consisting of 4 technical repeats). Features that are not selected by the machine learning pipeline have been colored grey (**b**). Values have been limited to between 1.5 and -0.4 for visualisation purposes only, the full range of data can be seen in Supplementary Figure 23a. The information for each sera sample can be found in Supplementary Table 11. Source data are provided as a Source Data file.

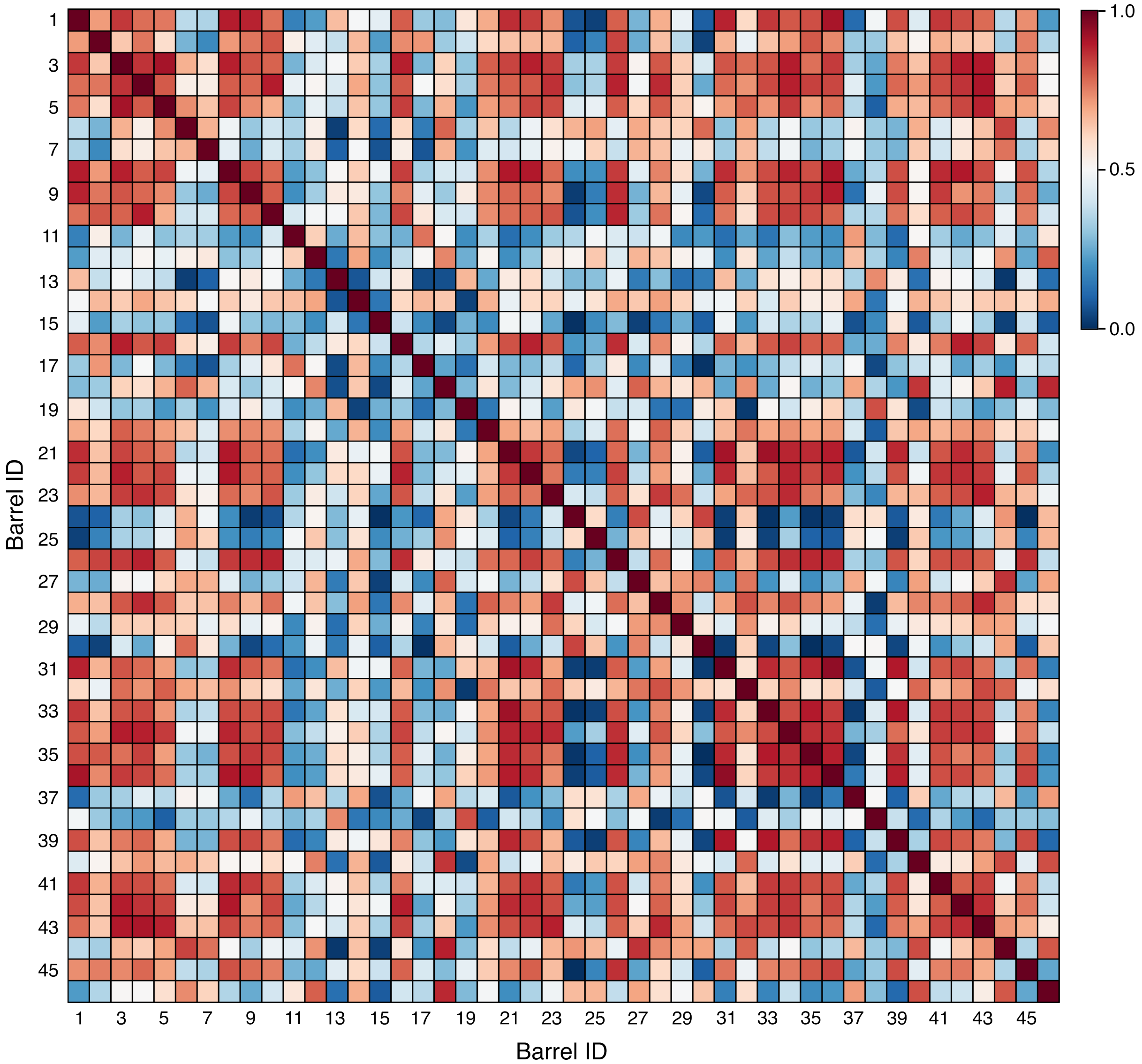

Supplementary Figure 21. Spearman coefficients of the αHBs in the αSA for the sera fingerprints. Color scheme is from strong correlation (dark red) to no correlation (dark blue) according to the heat map (right-hand side). Source data are provided as a Source Data file.

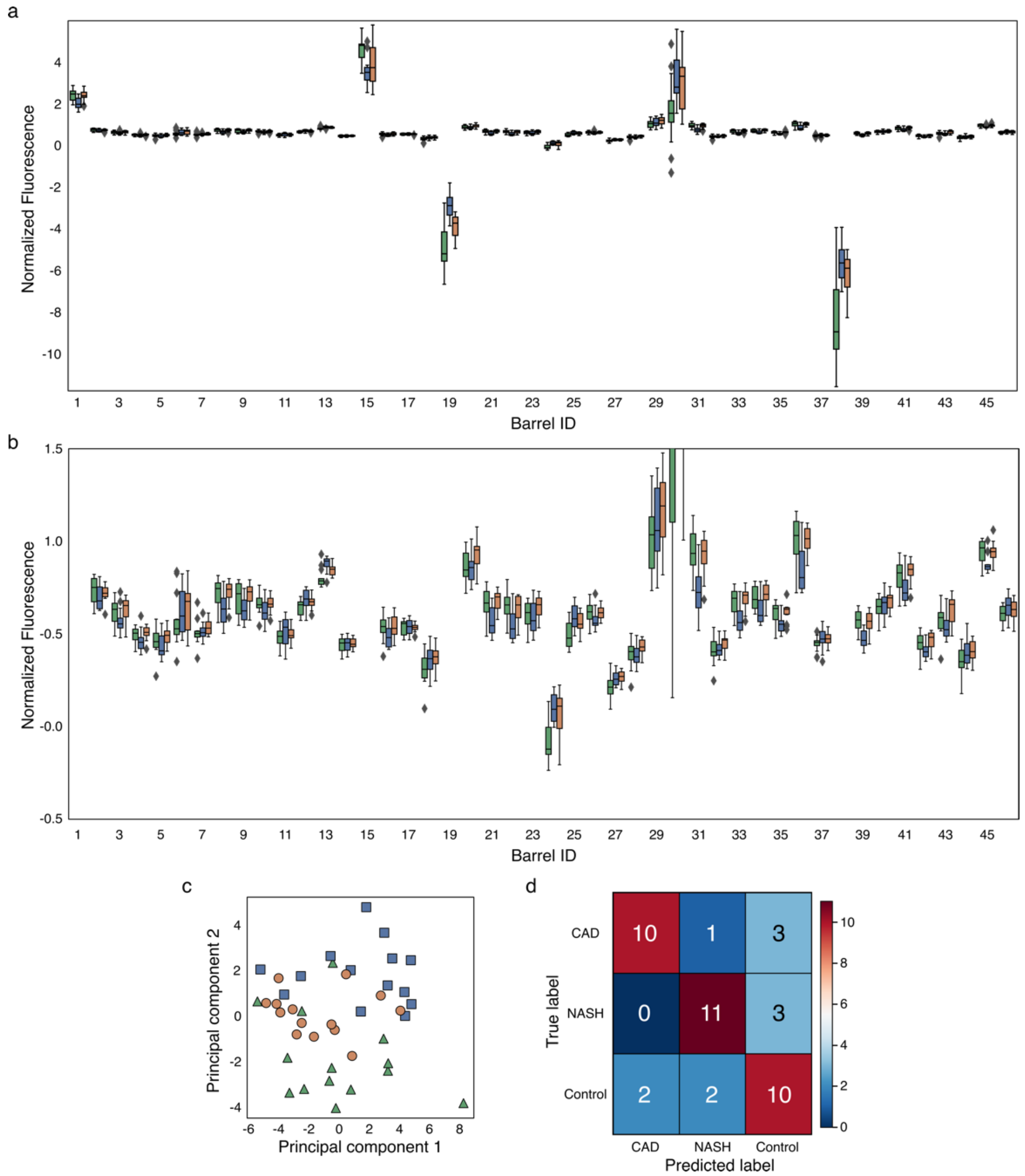

Supplementary Figure 22. αSA analysis for the differentiation of NASH, CAD and control sera samples. a & b, Full (**a**) and subsection (**b**) of min-max scaled fluorescent signals from the αSA challenged with different NASH and non-NASH sera samples (blue and orange respectively). Values are normalized relative to: 1, for the αHB and the reporter dye with no analyte; and 0, for the dye alone. Values between 1.5 and -0.5 are shown in (**b**) for clear visualization. Data corresponds to 42 independent samples that were measured 4 times to give a median value for each sera sample – n=14 NASH, n=14 CAD and n=14 control. Boxes show the interquartile range with the median presented as a line. Whiskers show 1.5 x interquartile range, or the range if a smaller value. Outliers are shown as diamonds. **c**, Principal component analysis of the 42 sera samples. NASH – blue square; CAD – green triangle; control – orange circle. **d**, Confusion matrix generated from predictions of NASH, CAD and control sera samples using LDA with nested cross-validation. The coloring scheme is from dark red (all prediction) to dark blue (no predictions) according to the heat map (right-hand side). Source data are provided as a Source Data file.

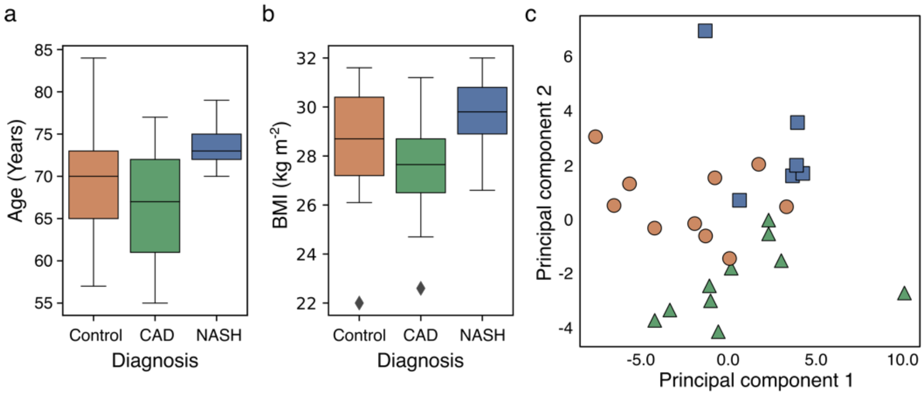

Supplementary Figure 23. Additional analysis of the sera from NASH, CAD and control patients. **a**, Age ranges of the NASH, CAD and control patients in the proof of concept study. Color scheme: NASH, blue; CAD, green; Control, orange. **b**, BMI ranges of the NASH, CAD and control patients in the proof-of-concept study. Color scheme same as in **a**. **a&b**, Data corresponds to 42 patients, n=14 NASH, n=14 CAD and n=14 control. Boxes show the interquartile range with the median presented as a line. Whiskers show maximum and minimum values. Outliers are shown as diamonds. **c**, Principal component analysis of non-obese (BMI<30) NASH, CAD and control patients' sera samples. Color scheme: NASH – blue squares, CAD – green triangles, control – orange circles. Source data are provided as a Source Data file.

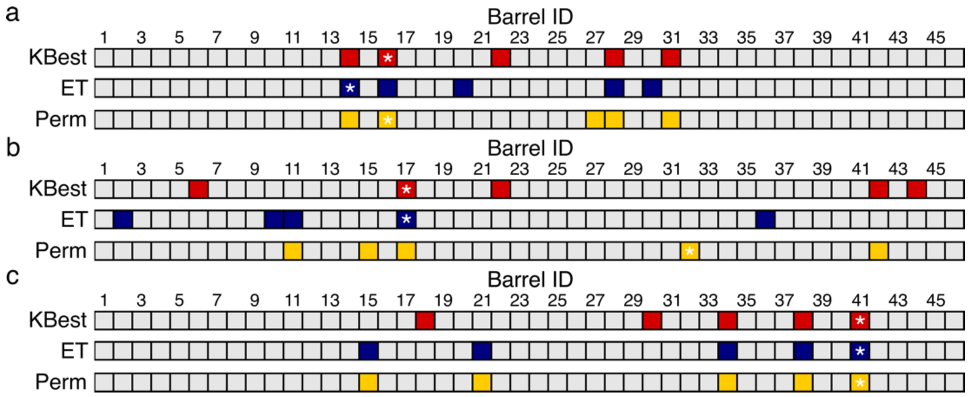

Supplementary Figure 24. Feature importance of the individual αHBs in the αSA. Feature importance of the αHBs in the classification of amino acids (**a**), fatty acids (**b**) and carbohydrates (**c**). The top five ranked αHBs calculated by KBest analysis, ExtraTrees (ET) and permutation analysis (Perm) are highlighted (red, blue and gold, respectively). The most important αHB determined by each method is marked (*).

References

1. Artrith, N. et al. Best practices in machine learning for chemistry. *Nat Chem* **13**, 505-508 (2021).
2. Rosner, B. Percentage Points for a Generalized ESD Many-Outlier Procedure. *Technometrics* **25**, 165-172 (1983).
3. Iglewicz, B. & Hoaglin, D.C. How to Detect and Handle Outliers. (ASQC Quality Press, 1993).
4. Fix, E. & Hodges, J.L. Discriminatory analysis. Nonparametric discrimination: Consistency properties. *Int Stat Rev* **57**, 238-247 (1989).
5. Steinley, D. K-means clustering: A half-century synthesis. *Br J Math Stat Psychol* **59**, 1-34 (2006).
6. Cortes, C. & Vapnik, V. Support-vector networks. *Mach Learn* **20**, 273-297 (1995).
7. Freund, Y. & Schapire, R.E. A Decision-Theoretic Generalization of On-Line Learning and an Application to Boosting. *J Comput Syst Sci* **55**, 119-139 (1997).
8. Pedregosa, F. et al. Scikit-learn: Machine learning in Python. *J Mach Learn Res* **12**, 2825-2830 (2011).
9. Kohavi, R. in Proceedings of the 14th international joint conference on Artificial intelligence - Volume 2 1137–1143 (Morgan Kaufmann Publishers Inc., Montreal, Quebec, Canada; 1995).
10. Dietterich, T.G. Approximate statistical tests for comparing supervised classification learning algorithms. *Neural Comput* **10**, 1895–1923 (1998).
11. Alpaydm, E. Combined 5 × 2 cv F Test for Comparing Supervised Classification Learning Algorithms. *Neural Comput* **11**, 1885-1892 (1999).
