# Supplementary material for: Differential sensing with arrays of de novo designed peptide assemblies
Source: Nat Commun. 2023 Jan 24;14:383. doi: 10.1038/s41467-023-36024-y (PMC9873944; doi:10.1038/s41467-023-36024-y)
Supplement: Supplementary file 3 — Description of additional Supplementary File [file 41467_2023_36024_MOESM3_ESM.pdf]

Descriptions of additional supplementary files

Supplementary Data File 1:
Sequences and associated data of all de novo designed alphaHBs used in this study

Supplementary Data File 2:
Merging and refinement statistics for the new X-ray crystal structures from this study
