# Supplementary material for: Differential sensing with arrays of de novo designed peptide assemblies
Source: Nat Commun. 2023 Jan 24;14:383. doi: 10.1038/s41467-023-36024-y (PMC9873944; doi:10.1038/s41467-023-36024-y)
Supplement: Supplementary file 6 — Reporting Summary [file 41467_2023_36024_MOESM6_ESM.pdf]

nature portfolio

Corresponding author(s): w.dawson@bristol.ac.uk, chris.wood@ed.ac.uk, d.n.woolfson@bristol.ac.uk

Last updated by author(s): Dec 12, 2022

Reporting Summary

Nature Portfolio wishes to improve the reproducibility of the work that we publish. This form provides structure for consistency and transparency in reporting. For further information on Nature Portfolio policies, see our Editorial Policies and the Editorial Policy Checklist.

Statistics

For all statistical analyses, confirm that the following items are present in the figure legend, table legend, main text, or Methods section.

n/a	Confirmed	
☐	☒	The exact sample size (*n*) for each experimental group/condition, given as a discrete number and unit of measurement
☐	☒	A statement on whether measurements were taken from distinct samples or whether the same sample was measured repeatedly
☐	☒	The statistical test(s) used AND whether they are one- or two-sided<br>*Only common tests should be described solely by name; describe more complex techniques in the Methods section.*
☒	☐	A description of all covariates tested
☒	☐	A description of any assumptions or corrections, such as tests of normality and adjustment for multiple comparisons
☐	☒	A full description of the statistical parameters including central tendency (e.g. means) or other basic estimates (e.g. regression coefficient) AND variation (e.g. standard deviation) or associated estimates of uncertainty (e.g. confidence intervals)
☐	☒	For null hypothesis testing, the test statistic (e.g. *F*, *t*, *r*) with confidence intervals, effect sizes, degrees of freedom and *P* value noted<br>*Give P values as exact values whenever suitable.*
☒	☐	For Bayesian analysis, information on the choice of priors and Markov chain Monte Carlo settings
☒	☐	For hierarchical and complex designs, identification of the appropriate level for tests and full reporting of outcomes
☒	☐	Estimates of effect sizes (e.g. Cohen's *d*, Pearson's *r*), indicating how they were calculated

Our web collection on statistics for biologists contains articles on many of the points above.

Software and code

Policy information about availability of computer code

Data collection	Circular dichroism: Spectra Manager (1.55). Analytical ultracentrifugation: ProteomeLab XL-A (5.5). Analyte/complex mixture assay: CLARIOstar, Software Version (5.40 R3).
Data analysis	Analytical ultracentrifugation - SEDFIT (v15.2b). X-ray diffraction data processing and model building: XIA2 (0.5.340-g5578c4a7-dials-1.6) pipeline (utilising AIMLESS (0.5.32), POINTLESS (1.11.1)), XSCALE (Build 20171111), XDS (Build 20200417), Dials (2.0.2), Phenix.phaser (2.8.3), Phenix (1.19.2_4158), CCP4 (7.1), REFMAC (5.8.0267), Coot (0.9.6). Machine learning code: imbalanced-learn (0.6.2), jinja2 (2.10.3), numpy (1.19.5), matplotlib (3.3.4), mlxtend (0.17.2), openpyxl (3.0.0), pandas (1.1.5), scikit-learn (0.24.1), scipy (1.5.4), seaborn (0.11.1), xlrd (1.2.0). All other data processed and plotted with Python (3.8.5), Numpy (1.19.2), matplotlib (3.1.3). Custom code written for the machine learning pipeline, and all data analysis, is available at https://github.com/woolfson-group/array_sensing and https://github.com/woolfson-group/array_sensing_data_analysis.

For manuscripts utilizing custom algorithms or software that are central to the research but not yet described in published literature, software must be made available to editors and reviewers. We strongly encourage code deposition in a community repository (e.g. GitHub). See the Nature Portfolio guidelines for submitting code & software for further information.

Data

Policy information about availability of data

All manuscripts must include a data availability statement. This statement should provide the following information, where applicable:

- Accession codes, unique identifiers, or web links for publicly available datasets
- A description of any restrictions on data availability
- For clinical datasets or third party data, please ensure that the statement adheres to our policy

All data is available. X-ray crystallography data for all assemblies has been publicly released onto the PDB with the following accession codes: 7NFF, 7NFG, 7NFH, 7FNI, 7NFJ, 7NFK, 7NFL, 7NFM, 7NFN, 7NFO, 7NFP, 8A09. Previously published X-ray crystallography data on the PDB is available with the following accession codes: 3R3K, 4PNA, 4PN8, 4PN9, 6EIK, 6G65, 6G66, 6G67, 6G6G.

Human research participants

Policy information about studies involving human research participants and Sex and Gender in Research.

Reporting on sex and gender	All serum samples were collected from female donors. No sex or gender based analysis was performed. The purpose and conclusions of this study are to investigate the performance of de novo peptide assemblies in a differential sensing technology rather than the analysis/diagnosis/response of a population.
Population characteristics	Serum samples were purchased with as similar as possible age and BMI for the NASH, CAD and control samples. No other covariates were considered. No covariates were analysed during the study.
Recruitment	Serum samples from donors with NASH, CAD and corresponding controls were purchased from the commercial biobank Proteogenex Inc. The protocols for obtaining samples were approved by the Ethics committee of the host organisation (PG-ONC 2003/1, 9/1/2020), with all donors signing informed consent documentation.
Ethics oversight	Serum samples from donors with NASH, CAD and corresponding controls were purchased from the commercial biobank Proteogenex Inc. The protocols for obtaining samples were approved by the Ethics committee of the host organisation (PG-ONC 2003/1, 9/1/2020), with all donors signing informed consent documentation.

Note that full information on the approval of the study protocol must also be provided in the manuscript.

Field-specific reporting

Please select the one below that is the best fit for your research. If you are not sure, read the appropriate sections before making your selection.

☒ Life sciences ☐ Behavioural & social sciences ☐ Ecological, evolutionary & environmental sciences

For a reference copy of the document with all sections, see nature.com/documents/nr-reporting-summary-flat.pdf

Life sciences study design

All studies must disclose on these points even when the disclosure is negative.

Sample size	No statistical method was used to determine sample size. A general approach that the number of data points, i.e. samples for the assay, should be approximately 10 times greater (as a minimum) than the number of categories in the classification was used for the small molecules. The maximum number of tea samples that could easily be obtained (30 different brands) were used, and the maximum of commercial sera samples within the budget was used. All initial sample size gave satisfactory datasets for machine learning applications and hence were not increased in size.
Data exclusions	All samples were analyzed for outliers within repeats using a generalized ESD test. This is a quality control measure that compares technical repeats within the same sample and hence analyzes errors in the assay setup/performance (e.g., liquid handling errors, or multi-well plate preparation errors) rather than the sample itself. 3 amino acid data points were excluded (2 x glutamate, 1 x arginine, 1 x serine, 1 x tryptophan), 5 fatty acid data points were excluded (2 x palmitic acid, 1 x oleic acid, 2 x nervonic acid) and 2 sugar datapoints were excluded (1x glucosamine, 1 x maltose) from the small molecule data. 2 tea datapoints were excluded (1 x black tea, 1 x green tea). No samples were excluded in the 3-way NASH-CAD-control analysis, 1 sample was excluded in the 2-way NASH-No-NASH analysis (1 x No-NASH). These details are also available in the Methods section.<br>No samples were excluded for any other reasons.
Replication	All attempts at replication were successful although see above for subsequent data exclusions. Small molecule analytes underwent 10 independent measurements (n=10, 50 datapoints for each class). Each of the 30 tea brands was independently replicated 6 times (n=6, 180 datapoints). The 42 independent sera samples underwent 4 technical replicates that were combined to give the median measurement for each of the sera samples (42 datapoints).
Randomization	Commercial sera samples were purchased with a diagnoses of NASH (& CAD), CAD or neither. No other covariates were analyzed in this study

as it is analyzing the performance of the sensor technology - the de novo peptide assembly array - rather than the signal from different groups of individuals.

Blinding	A specific hypothesis tested by statistics is not being investigated in this study, so blinding is not applicable. The study investigates whether de novo peptide assemblies can be used in differential sensing technology from samples with known differences.

Reporting for specific materials, systems and methods

We require information from authors about some types of materials, experimental systems and methods used in many studies. Here, indicate whether each material, system or method listed is relevant to your study. If you are not sure if a list item applies to your research, read the appropriate section before selecting a response.

Materials & experimental systems

n/a	Involved in the study	
☒	☐	Antibodies
☒	☐	Eukaryotic cell lines
☒	☐	Palaeontology and archaeology
☒	☐	Animals and other organisms
☒	☐	Clinical data
☒	☐	Dual use research of concern

Methods

n/a	Involved in the study	
☒	☐	ChIP-seq
☒	☐	Flow cytometry
☒	☐	MRI-based neuroimaging
